# Transcriptomic analysis to infer key molecular players involved during host response to NDV challenge in *Gallus gallus* (Leghorn & Fayoumi)

**DOI:** 10.1038/s41598-021-88029-6

**Published:** 2021-04-19

**Authors:** Venkata Krishna Vanamamalai, Priyanka Garg, Gautham Kolluri, Ravi Kumar Gandham, Itishree Jali, Shailesh Sharma

**Affiliations:** 1 National Institute of Animal Biotechnology (NIAB), Opposite Journalist Colony, Near Gowlidoddi Extended Q City Road, Gachibowli, Hyderabad, Telangana 500032 India; 2 ICAR-Central Avian Research Institute, Izatnagar, Bareilly, Uttar Pradesh 243122 India

**Keywords:** Animal biotechnology, Computational biology and bioinformatics, Genome informatics

## Abstract

Long non-coding RNAs (lncRNAs) are the transcripts of length longer than 200 nucleotides. They are involved in the regulation of various biological activities. Leghorn and Fayoumi breeds of *Gallus gallus* were known to be having differential resistance against Newcastle Disease Virus (NDV) infection. Differentially expressed genes which were thought to be involved in this pattern of resistance were already studied. Here we report the analysis of the transcriptomic data of Harderian gland of *Gallus gallus* for studying the lncRNAs involved in regulation of these genes. Using bioinformatics approaches, a total of 37,411 lncRNAs were extracted and 359 lncRNAs were differentially expressing. Functional annotation using co-expression analysis revealed the involvement of lncRNAs in the regulation of various pathways. We also identified 1232 quantitative trait loci (QTLs) associated with the genes interacting with lncRNA. Additionally, we identified the role of lncRNAs as putative micro RNA precursors, and the interaction of differentially expressed Genes with transcription factors and micro RNAs. Our study revealed the role of lncRNAs during host response against NDV infection which would facilitate future experiments in unravelling regulatory mechanisms of development in the genetic improvement of the susceptible breeds of *Gallus gallus*.

## Introduction

RNA-seq generated transcriptomic data was being used across the world to study the contrasts in expression profile^1^. Recent studies are showing that long non-coding RNA are involved in various cellular activities and have potential to contribute towards disease resistance mechanisms. Long non-coding RNAs are the RNA molecules of length greater than 200 and are transcribed by RNA Polymerase II, same as messenger RNA. Similar to messenger RNA, many lncRNA contain a 5′ cap and some lncRNA can also have a 3′ poly-A tail. Some lncRNAs contain introns and undergo splicing similar to messenger RNA^2^. Although they do not encode any proteins, they are involved in various cellular activities. They are found in nuclear, cytoplasmic and other cellular compartments. They are relatively unstable and are poorly conserved, while a few are known to be conserved^3^.

Long non-coding RNAs are involved in various activities like chromatin modification, molecular scaffolding, reduction of micro RNA activity by complementary base pairing (Sponging effect), enhancement or suppression of the gene expression by guiding transcription factors to the promoter sequences or by preventing them from binding, alteration of splicing patterns of genes or their degradation similar to micro RNA^4^. Recent research showed that lncRNAs mediate disease pathogenesis and thus challenge the concept that protein coding genes are the only contributors to development of diseases^5^.

Long non-coding RNAs emerged as a major category of regulatory eukaryotic transcripts and can be classified into different groups based on their location within a genome. Long intergenic RNAs (linc RNAs) are the transcripts in intergenic regions. Intronic lncRNAs are the transcripts located in the intronic region of protein coding genes. Sense lncRNAs are the transcripts located on the sense strand of protein coding genes including both exons and introns of those genes and Anti-sense lncRNAs are the transcripts located on the anti-sense strand of protein coding genes^6^. LncRNAs are initially discovered in bacteria in 1980s and the sequencing of the human genome has revealed tens of thousands of lncRNAs from each class that are transcribed from the specific cell and tissue types^7^. Although relatively few lncRNAs have been functionally characterized, increasing evidence suggests an important role of these transcripts.

Poultry is most commonly available, cheapest and acceptable source of protein that accounts for more than 30% in the livestock. Indian poultry industry has made a fastest and remarkable growth ever since its inception and is presently emerging sector with a growth rate of 12–15%, posting an annual turnover of 10,000 million dollars and also satisfying the hungers of 20 million people through employment.

New castle disease virus is one of the serious threats to global poultry industry. Previous studies have shown that Fayoumi breed is relatively resistant to NDV compared to Leghorn breed^8^. Today’s chicken are considered to be the descendant of Red Jungle Fowl. Leghorn and Fayoumi breeds of chicken have differential resistance against Newcastle Disease Virus. It is thought that, in Leghorn breed, the alleles conferring resistance might have lost or mutated during this course. The resistant Fayoumi breed has been reported to contain more viral transcripts at initial stage of infection than the susceptible Leghorn breed and clear the virus load and get rid of the infection by quickly, while the susceptible Leghorn breed contains less virus load initially but gets severe NDV infection and most of the birds die by latter stages of infection^10^. Till date, several studies are performed for the genes in various tissues like Trachea^8^, Lungs^9^, Harderian gland^10^ etc. But the role of lncRNA is not yet studied. The objective of the present study is the analysis of the host responses against New castle disease virus in Leghorn and Fayoumi breeds of *Gallus gallus domesticus* for understanding the role of long non-coding RNA in the NDV disease resistance.

## Results

### Pre-processing of RNA-seq data

The data of 94 samples downloaded from EBI-ENA database was about 424 GB. The 94 samples are of Leghorn and Fayoumi breeds at three different time points i.e., 2 DPC, 6 DPC and 10 DPC. The quality control by FastQC^11^ showed that sequence data contain Illumina adapters. Adapter trimming was performed by using Trimmomatic tool v0.38^12^. Among all the 94 samples, percentage of surviving reads ranged between 75 and 99%. The number of input raw reads and surviving reads of each sample was mentioned in Supplementary Table S1. After adapter trimming, FastQC tool^11^ results showed that 48 Leghorn sample sequences contain 47% GC content on average and per base quality scores range from 24 to 33 and 46 Fayoumi sequences contain 46% GC content on average and per base quality scores range from 26 to 33. The results were merged using MultiQC v1.7^13^. The percentage of duplicates, GC content and total sequences of each sample were mentioned in Supplementary Table S2.

### Differential expression analysis

The samples showed an average mapping percentage of 85% against the reference genome of *Gallus gallus* (GRCg6a). One of the Fayoumi 2 DPC samples was discarded as it showed mapping percentage of 10%, indicating technical failure as mentioned in the study by Iowa State University^10^. There was a great variability in number of transcripts in each DPC when assembly was performed using Reference annotation (GRCg6a) than by using the merged file as annotation with Hisat 2^14^. The percentage of mapping and number of transcripts in each sample were mentioned in Supplementary Table S3. As per the new Tuxedo pipeline^15^, the transcripts files were processed by the python script provided with Stringtie^16^ and two files namely, gene count matrix and transcript count matrix were obtained, which contain the number of reads per each gene and transcript respectively. The gene read count matrix was used to estimate the differential expression by using edgeR^17^. Several differentially expressed genes and lncRNAs were obtained in each DPC which include both up-regulated and down-regulated sequences (Table 1) and were shown in form of heat maps in Supplementary Figures S1 and S2 respectively.

**Table 1:** Number of differential expressed genes (DEGs) and long non-coding RNAs (DE-lncs) of Leghorn and fayoumi at each time points.

Breed	Leghorn		Fayoumi	
DPC	DEGS	DE-lncs	DEGS	DE-lncs
2 DPC	4	1	4	23
6 DPC	424	251	12	6
10 DPC	10	3	58	75

**Figure 1:**
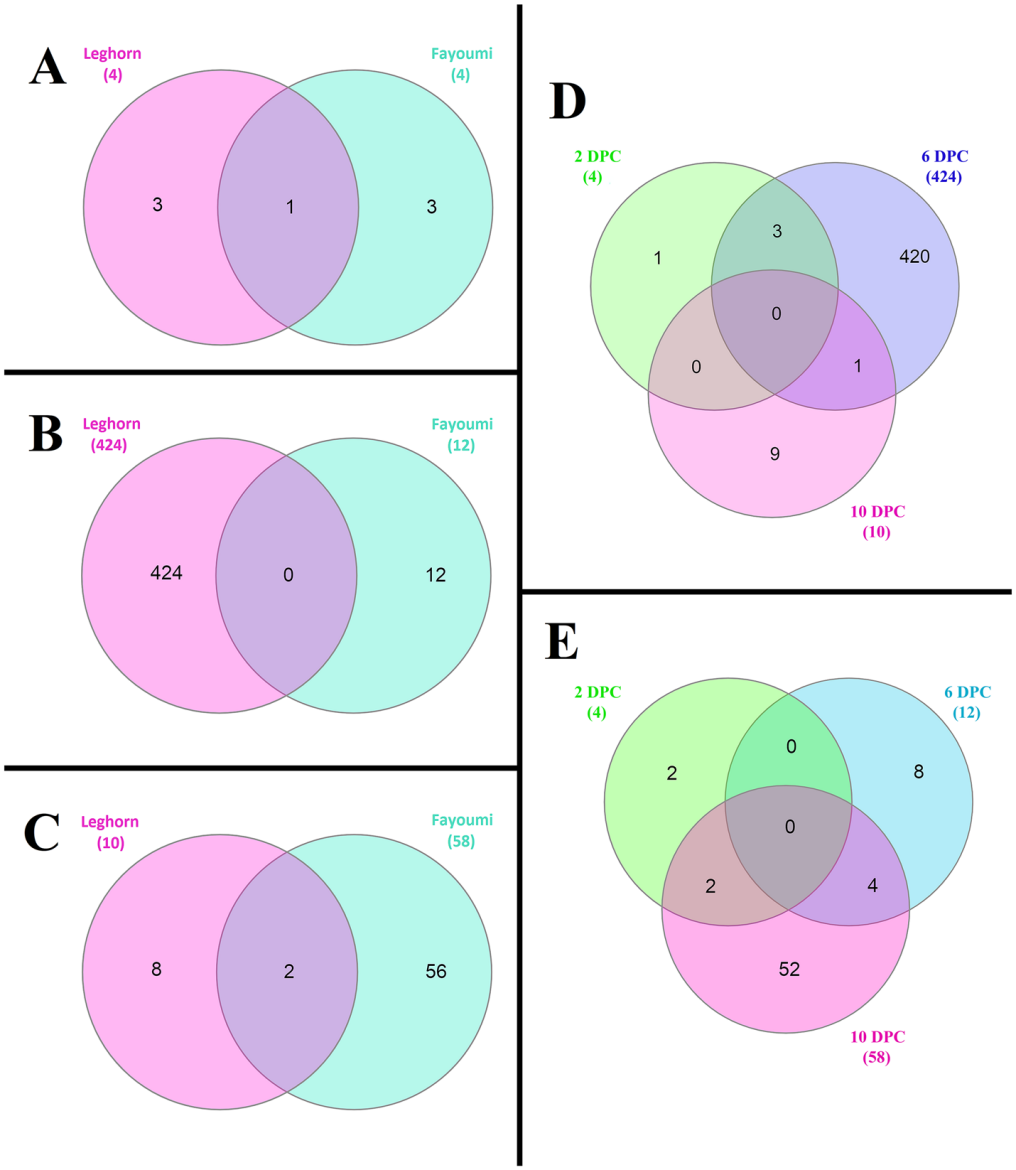
Differential expression analysis. Venn diagrams showing common and unique differentially expressed genes of Leghorn and Fayoumi at 2 DPC (**A**), 6 DPC (**B**) and 10 DPC (**C**), within each time point of Leghorn (**D**) and Fayoumi (**E**) plotted by InteractiVenn.

**Figure 2:**
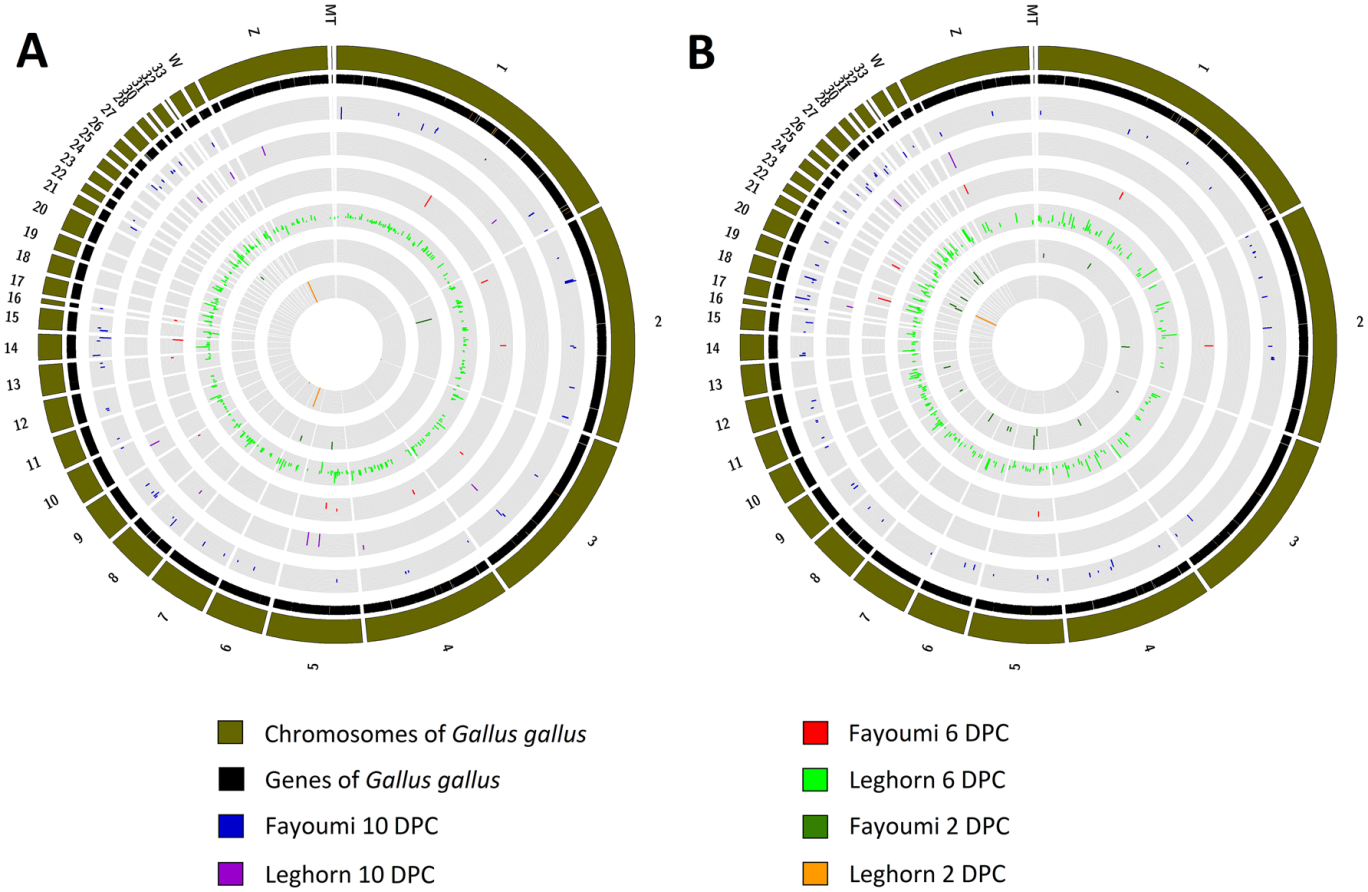
Differential expression analysis. Circos figure showing the chromosomal localization of differentially expressed genes (**A**) and long non-coding RNAs (**B**) of Leghorn and Fayoumi at 2 DPC, 6 DPC and 10 DPC.

The Venn diagrams obtained by using InteractiVenn^18^ showed that there were very few common DEGs between Leghorn and Fayoumi at each DPC and there were no common DEGs between different time points of the same breed. This shows that at each time point, Leghorn and Fayoumi were expressing different and unique genes. Even between the breeds, different genes were expressed at different time points, there is only 1 common DEG at 2 DPC, no common at 6 DPC and 2 common at 10 DPC. (Fig. 1). The Circos^19^ plots (Fig. 2) show the localization of the DEGs (A) and DElncRNAs (B). From the plot, we can conclude that there were more number of DEGs and DElncRNAs in Leghorn 6 DPC while least in Leghorn 2 DPC. In case of DEGs, at 2 DPC Leghorn and Fayoumi, 4 DEGs were found on 4 different chromosomes. At Leghorn 6 DPC and Fayoumi 10 DPC, most number of DEGs were found on chromosome 1, on chromosome 5 in Leghorn 10 DPC and on chromosome 2 on Fayoumi 6 DPC. While in case of DElncRNAs, in Leghorn 2 DPC, only 1 DElncRNA was obtained and was on Chromosome 20, in Leghorn 6 DPC, most number of DElncRNAs were observed on Chromosome 1, in Leghorn 10 DPC, 3 different DElncRNAs were found on 3 different chromosomes, in Fayoumi 2 DPC, most number of DElncRNAs were found on chromosomes 1, 5, 6, 20, 27, 28, in Fayoumi 6 DPC, 6 different DElncRNAs were found on 6 different chromosomes and in Fayoumi 10 DPC, most number of DElncRNAs were observed on Chromosome 2.

Simple sequence repeats (SSRs) in DEGs and DElncRNAs were identified by using MISA^20^ standalone tool version 2.0 separately for each DPC. Different types of SSRs were reported, including compound C, mononucleotide, dinucleotide, trinucleotide, tetranucleotide, pentanucleotide and hexanucleotide SSRs. The chromosomal localization of these SSRs was plotted by Circos^19^ plot (Supplementary Figure S3). From the plot, we can conclude that there were more number of SSRs in the DEGs and differentially expressed lncRNAs of Leghorn 6 DPC and Fayoumi 10 DPC. At 2 DPC, there were more number of SSRs in DEGs of Fayoumi. In case of DElncRNAs, highest number of SSRs were found in Leghorn 6 DPC, followed by Fayoumi 10 DPC, while there were no SSRs obtained in Leghorn 2 DPC.

### Genome-wide identification of long non-coding RNAs in *Gallus gallus*

**Figure 3:**
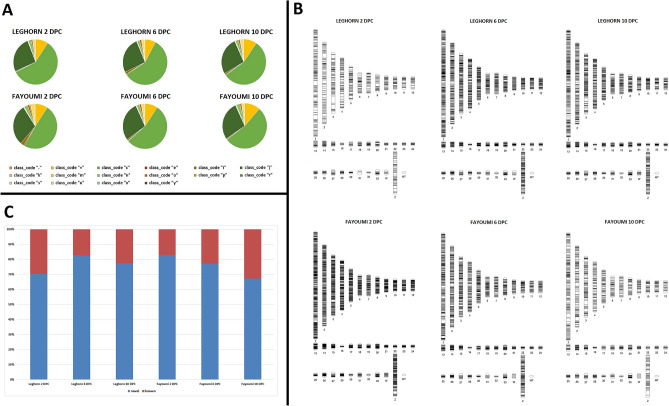
Genome-wide Identification of long non-coding RNAs. (**A**) Piecharts showing the different class codes of Leghorn and Fayoumi at 2 DPC, 6 DPC and 10 DPC. (**B**) Figure showing the chromosomal localization of long non-coding RNAs of Leghorn and Fayoumi at 2 DPC, 6 DPC and 10 DPC plotted by Phenogram. (**C**) Figure showing histograms of known and novel long non-coding RNAs of Leghorn and Fayoumi at 2 DPC, 6 DPC and 10 DPC.

There were various number of transcripts assigned to each of the 16 class codes^21^. From Fig. 3A, we can conclude that more than half of the transcripts were assigned to class code “c” (intron compatible) while very few transcripts were assigned to class code “.” and “s” (mapping errors). The sequences of class codes of “i” (intronic), “u” (intergenic/unknown), “x” (antisense transcript) were extracted using Bedtools^22^. After filtering through various steps, there were a greater number of lncRNAs observed in Fayoumi than in Leghorn with highest number of lncRNAs in Fayoumi 2 DPC (12,005) and least in Fayoumi 10 DPC (3454) (Table 2).

**Table 2:** Number of transcripts at each step in long non-coding RNA extraction of Leghorn and Fayoumi at each time points.

Breeds	Leghorn			Fayoumi		
Step	2 DPC	6 DPC	10 DPC	2 DPC	6 DPC	10 DPC
Total (IUX)	5034	8512	7618	14,189	7294	5060
Len filter (> 200)	4611	8220	7357	13,687	6781	4622
ORF filter (< 300)	4282	7848	6843	13,080	6399	4214
CPC2 (noncoding)	3741	7289	6201	12,308	5772	3684
Pfam (no hits)	3932	7453	6469	12,588	5991	3907
Noncoding + no hits	3486	6981	5902	11,908	5464	3448
No ORF	4	55	39	97	21	6
Total	3490	7036	5941	12,005	5485	3454

### Characteristics of the identified long non-coding RNAs

Different characteristics of the predicted lncRNAs were examined (Supplementary Table S4).

- **Classification of lncRNAs:**
  The long non-coding RNAs were classified basing on their expression values (log fold change) in different samples as highly up regulated (> 4), normally up regulated (0–4), highly down regulated (< − 2) and normally down regulated (0 to − 2). In Leghorn, on an average, about 79% of the lncRNAs were found to be upregulated and about 21% were found to be down regulated, while in Fayoumi, about 55% of lncRNAs were found to be upregulated and about 45% were found to be down regulated. On an average, most of the lncRNAs in both leghorn and Fayoumi were of normally up regulated category, while a greater number of highly down regulated lncRNAs were found in Fayoumi.
- **Chromosomal distribution**
  The analysis of distribution of all the lncRNAs predicted across the two breeds and three time points showed that they were not evenly distributed across the 35 chromosomes. The localization of the extracted lncRNAs plotted by Phenogram^23^ shows that the highest proportion of lncRNAs (16%) were located on chromosome 1 and the lowest proportion (0.2%) on chromosome W (Fig. 3B).
- **Length distribution**
  The length of the extracted lncRNAs ranged from 301 bases to 275,367 bases, with more than half (55.99%) between 1000 and 10,000 bases. On contrast, the length of coding transcripts (class code e) ranged from 199 bases to 15,638 bases, indicating that the long non-coding transcripts are longer than coding transcripts.
- **Noncode database**
  The BlastN^24^ search against known lncRNAs of *Gallus gallus* on noncode database version 5.0^25^ showed several known and novel lncRNAs in each DPC of Leghorn and Fayoumi. Most of the novel lncRNAs (80%) were found in Leghorn 6 DPC and Fayoumi 2 DPC, while least number (65%) was observed in Fayoumi 10 DPC (Fig. 3C).
- **Transfer RNA and ribosomal RNA databases**
  The BlastN^24^ search against transfer RNA^26^ and ribosomal RNA database^27,28^ showed no similarities in all DPCs of both Leghorn and Fayoumi.
- **MicroRNA database (mirBase)**
  The BlastN^24^ search showed no similarities in all DPCs against mature miRNAs but several lncRNAs matched to hairpin miRNAs showing them as potential precursors of those miRNAs on mirBase^29^. Most of matches were found in Fayoumi 2 DPC and least in Leghorn 2 DPC and Fayoumi 10 DPC (Table 3).

**Table 3:** Number of matches of long non-coding RNAs with hairpin micro-RNA from mirBase of Leghorn and Fayoumi at each time points.

Breeds	Leghorn			Fayoumi		
Step	2 DPC	6 DPC	10 DPC	2 DPC	6 DPC	10 DPC
Total	3490	7036	5941	12,005	5485	3454
Matches	31	38	25	41	32	31

### GO enrichment analysis

Gene Ontology Analysis was performed by Blast2GO^35^ and the data distribution chart shows that less than 0.05% of DEGs have no significant blast hits and more half of the DEGs were annotated with a GO term (Supplementary Figure S4) and about 0.4% of DElncRNAs have no significant blast hits and about 0.3% of the DElncRNAs were annotated with a GO term (Supplementary Figure S5). GO terms for biological process (BP), molecular function (MF) and Cellular component (CC) for both DEGs and DElncRNAs were obtained and mentioned in Supplementary Tables S5 and S6 respectively. For BP in DEGs, GO terms up to level 9 were reported, for MF, GO terms up to level 9 were reported and for CC, GO terms up to level 7 were reported. In case of DElncRNAs, for BP, GO terms up to level 8 were reported, for MF, GO terms up to level 8 and for CC, GO terms up to level 6 were reported. In all the datasets, more than 75% of the GO IDs were for BP, 13% for MF and 12% for CC. Most number of GO terms were obtained for Fayoumi 6 DPC, while least number for Leghorn 10 DPC. The number of GO terms obtained in each time point for DEGs and DElncRNAs of both the breeds was mentioned in Table 4.

**Table 4:** Number of GO terms for differentially expressed genes (A) and differentially expressed lncRNAs (B) of Leghorn and Fayoumi at each time point.

Breed	Leghorn			Fayoumi		
DPC	2 DPC	6 DPC	10 DPC	2 DPC	6 DPC	10 DPC
**(A)**						
BP	108	101	36	111	585	190
MF	30	22	15	26	76	30
CC	17	25	27	17	52	39
**(B)**						
BP	90	130	0	190	97	148
MF	6	26	0	26	20	25
CC	15	35	0	30	20	66

In KEGG pathway analysis of Leghorn, no pathways were reported at 2 DPC, 54 pathways were reported in 6 DPC and 4 pathways were reported at 10 DPC while in Fayoumi, only 1 pathway was reported at 2DPC, no pathways were reported at 6 DPC and 5 pathways were reported at 10 DPC. In Fayoumi, very few numbers of KEGG pathways were reported compared to Leghorn. These are mentioned in Supplementary Table S5.

### Co-expression analysis of the lncRNAs

**Figure 4:**
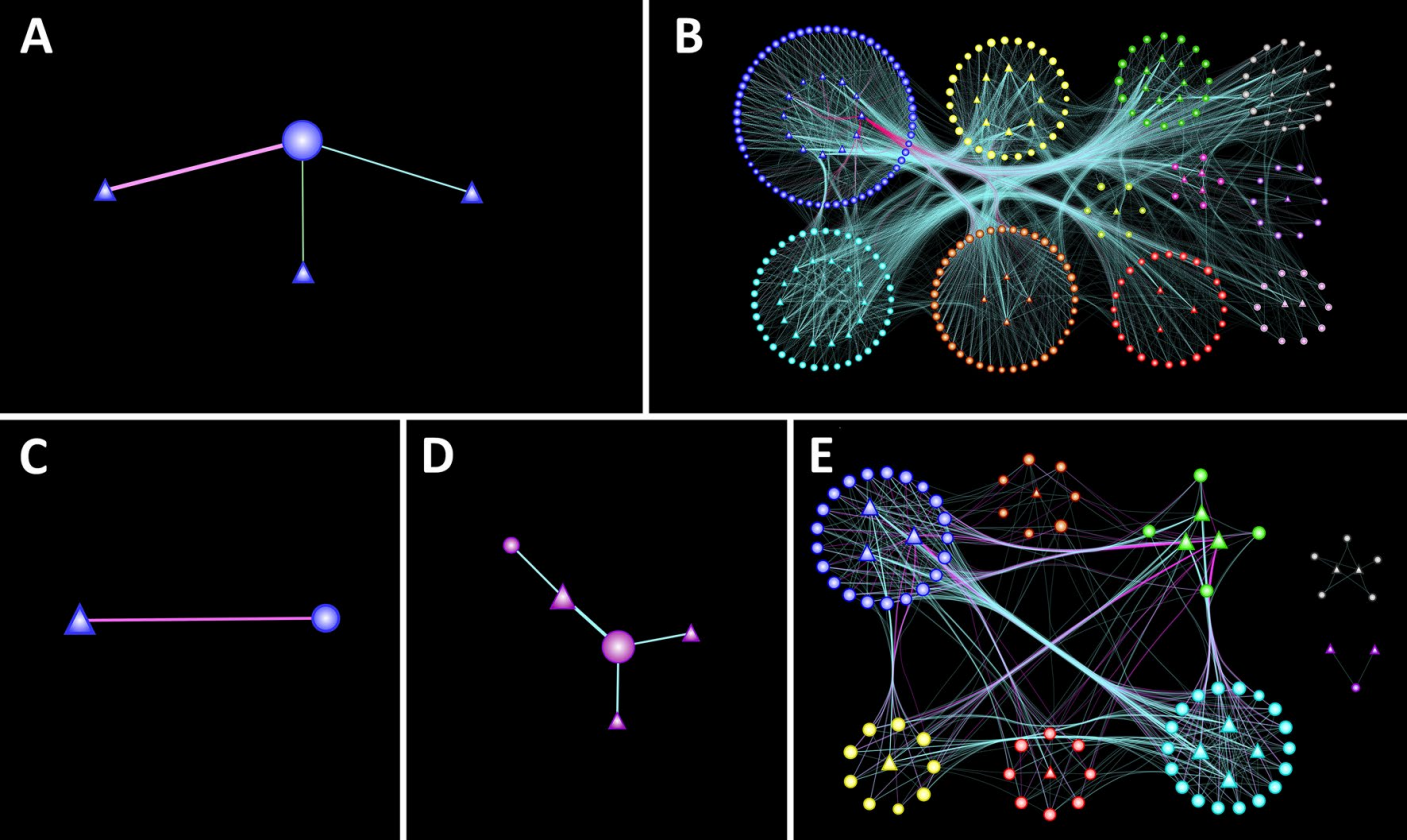
Figure showing networks of differentially expressed genes and long non-coding RNAs of Leghorn 2 DPC (**A**), 6 DPC (**B**) and Fayoumi 2 DPC (**C**), 6 DPC (**D**) and 10 DPC (**E**) plotted using Cytoscape v3.8.2 (https://cytoscape.org/). Triangle—Gene, Ellipse—lncRNA, Node colours—Modules, Edge colours (Blue—Cellular Process (GO:0009987), Green—Immune system development (GO:0002520), Pink—Response to stress (GO:0006950)).

The co-expression analysis of the lncRNAs with genes by using WGCNA^30^ showed that there were a greater number of interactions in Leghorn 6 DPC and Fayoumi 10 DPC. The modules of interaction were showed with different colours for all time points in Supplementary Figure S6. The presence of many modules of interactions with a greater number of genes and lncRNAs between differentially expressed lncRNAs and DEGs at Leghorn 6 DPC and Fayoumi 10 DPC indicate multiple pathways getting regulated at these time points. The co-expression networks plotted using Cytoscape v3.8.2^31^ shows the interactions between lncRNAs and genes. The network plot in Fig. 4 shows the interactions between lncRNAs and genes involved in biological processes—Cellular Process (GO:0009987), response to stress (GO:0006950) and Immune system development (GO:0002520), which were obtained from Blast2GO. The different colours of edges indicate different Biological process, thickness of edges is based on the weight, different colour of nodes indicate different modules of interaction and different shapes of nodes indicate genes and lncRNAs. The figure shows that the greatest number of interactions were observed between lncRNAs and genes including within the modules and between different modules in Leghorn 6 DPC and Fayoumi 10 DPC. While Leghorn 2 DPC, Fayoumi 2 DPC and 6 DPC had only single module of interaction and Leghorn 10 DPC had no interaction in the selected Biological processes. The network plot with the interactions including all the BPs at all the time points was shown in Supplementary Figure S7. It is also observed that the lncRNAs were interacting with different genes of different modules involved in different Biological processes. Further the potential functions of differentially expressed lncRNAs were predicted by determining the functions of the co-expressing DEGs.

### Cis and Trans regulation

Across all the six conditions (Breeds and time points), about 93% of all the gene and lncRNA interactions were observed to be trans-regulatory, while only 7% were cis-regulatory. The interactions along with the chromosomal localization were plotted using Circos^19^, and the plot is shown in Supplementary Figure S8. This plot clearly shows that there more trans regulatory interactions than cis. Highest number of interactions were found in Leghorn 6 DPC, while lowest number of interactions were found in Fayoumi 2 DPC. The number of interactions in each condition was mentioned in Table 5.

**Table 5:** Number of cis and trans regulatory lncRNA-gene interactions of Leghorn and Fayoumi at each time point.

Breed	Leghorn			Fayoumi		
DPC	Total	Cis	Trans	Total	Cis	Trans
2 DPC	3	0	3	2	1	1
6 DPC	50,172	3172	47,000	6	1	5
10 DPC	5	0	5	2430	117	2313

### Functional analysis of lncRNAs

The potential functions of the differentially expressing lncRNAs were predicted by co-expression analysis (Supplementary Table S7). There were 3 interactions predicted to be involved in 3 pathways in Leghorn 2 DPC, 22,026 interactions predicted to be involved in 176 pathways in Leghorn 6 DPC, 6 interactions predicted to be involved in 2 unknown pathways in Leghorn 10 DPC, 2 interactions predicted to be involved in 2 pathways in Fayoumi 2 DPC, 7 interactions predicted to be involved in 4 pathways in Fayoumi 6 DPC and 2136 interactions predicted to be involved in 48 pathways in Fayoumi 10 DPC. In Leghorn 2 DPC, 1 lncRNA was interacting with 1 novel gene, in Leghorn 6 DPC, the 131 lncRNA were interacting a single novel gene, in Leghorn 10 DPC, 3 lncRNAs were interacting with 2 novel genes, while in Fayoumi 2 DPC, 1 lncRNA was interacting with 1 novel gene, in Fayoumi 6 DPC, 1 lncRNA was interacting with 1 novel gene and in Fayoumi 10 DPC, 4 lncRNA were interacting with 15 novel genes.

At 2 DPC in leghorn, lncRNAs were reported to be up regulating the genes involved in Defence response to virus and lymphoid progenitor cell differentiation, while in Fayoumi, the lncRNAs were found to be up regulating the genes involved in defence response to virus and negative regulation of protein processing. At 6 DPC in Leghorn, although there were more number of interactions, most of lncRNAs were reported to be co-expressing with genes involved in metabolic and biosynthetic processes, and a few were found to be involved in signalling pathways related to immunological processes, while in Fayoumi, the lncRNAs were found to be up regulating the genes involved in metabolic and signalling pathways. Finally at 10 DPC in Leghorn, lncRNAs were reported to be up regulating some novel unknown genes, while in Fayoumi, the lncRNAs were found to be up regulating the genes involved in inflammatory processes and certain signalling pathways and down regulating the genes involved in the negative regulation of transcription. It is also observed that, across all the time points, most of the lncRNAs which were up regulated were co-expressing with the genes which also get up-regulated. Although there were certain genes with multiple lncRNAs getting co-expressed, in which few lncRNAs were in contrast. Thus, we can assume that these lncRNAs might be augmenting the expression of the genes which were co-expressed with them.

From this, we can conclude that, the absence of proper immune related genes and lncRNAs co-expressing with such genes could be the reason for the Leghorn breed being relatively susceptible to Newcastle Disease Virus than Fayoumi breed, which showed immune related genes and lncRNAs co-expressing with them.

### QTL (quantitative trait loci) analysis

The analysis of DEGs with QTL database^36^ revealed several DEGs that were associated with different QTLs. There were several types of QTLs including Exterior QTLs, Health QTLs, Physiology QTLs, Production QTLs and Reproduction QTLs. In health QTLs category, “Body temperature”, “Antibody titer to SRBC antigen”, “Fecal egg count”, “Antibody titer to IBD”, “Pullorum disease susceptibility” and “Antibody response to SRBC antigen” were reported. The number of DEGs reported to be associated with different QTLs in each condition was mentioned in Table 6. The details of all the QTLs associated with the DEGs were mentioned in Supplementary Table S8.

**Table 6:** Number of QTL types reported in Leghorn (2 DPC, 6 DPC and 10 DPC) and Fayoumi (2 DPC, 6 DPC and 10 DPC).

Time point	Total QTL	Exterior QTL	Health QTL	Physiology QTL	Production QTL	Reproduction QTL
Leghorn 2 DPC	5	0	0	0	5	0
Leghorn 6 DPC	1165	177	28	21	898	41
Leghorn 10 DPC	14	0	0	0	14	0
Fayoumi 2 DPC	4	0	0	0	4	0
Fayoumi 6 DPC	6	0	0	0	6	0
Fayoumi 10 DPC	38	4	0	0	32	2

### Interactions between DEGs and transcription factors

MEME^32^ identified different motifs in the 5′ upstream untranslated regions of the differentially expressed genes at all the time points in both breeds with a maximum cut-off of 10 motifs per gene. TomTom^32^ identified several potential transcription factors using these motifs. But all the transcription factors obtained were of different species like Human and Mouse. Thus, these transcription factors were searched against the known transcription factors of *Gallus gallus* in Animal Transcription factor database^33^. Different number of transcription factors were obtained in each time point of Leghorn and Fayoumi breeds (Table 6). Apart from that, transcription factors were identified for only 7 motifs at Leghorn 6 DPC and Fayoumi 10 DPC, 5 motifs at Fayoumi 6 DPC, 4 motifs at Leghorn 2 DPC and Leghorn 10 DPC, while at Fayoumi 2 DPC, transcription factors were identified for only 2 motifs. The obtained transcription factors were annotated using the annotation data from Animal Transcription factor database^33^, which was mentioned in Supplementary Table S9.

### Interactions between DEGs and microRNAs

Several numbers of potential miRNAs interacting with DEGs were identified using miRDB^34^. The total number of miRNAs interacting with each gene at each time point were mentioned in Table 7 and the top-most miRNA based on target score for each gene was mentioned in Supplementary Table S10. More than half of the DEGs of all the time points had no miRNAs—in Leghorn 2 DPC, 50% of DEGs, in Leghorn 6 DPC, 43% of DEGs, in Leghorn 10 DPC, 50% of DEGs, in Fayoumi 2 DPC, 75% of DEGs, in Fayoumi 6 DPC, 66% of DEGs and in Fayoumi 10 DPC, 50% DEGs had no miRNAs interacting with them.

**Table 7:** Number of transcription factors and miRNA interacting with DEGs of Leghorn and Fayoumi at each time point.

Breed	Leghorn			Fayoumi		
Time point	2 DPC	6 DPC	10 DPC	2 DPC	6 DPC	10 DPC
DEGs	4	424	10	4	12	58
Transcription factors interacting with DEGs	36	67	41	44	46	66
Motifs with Transcription Factors	4	7	4	2	5	7
miRNA interacting with DEGs	85	15,384	485	140	640	2327

### Sequence similarity analysis

The extracted lncRNAs of all six conditions were merged and analysed for similarity by using Blast^24^ search against the lncRNA data of all the available species on Noncode database^25^. Most number of 100% matches were obtained against Mouse (37 matches), followed by Human (11), *Drosophila melanogaster* (6), Opossum (6), Rat (4), Rhesus monkey (4), Zebra fish (3), Pig (3), Chimp (2) and Gorilla (2). Only 1 match was obtained against *C. elegans*, Cow, Orangutan and Platypus. There were no 100% similar matches against *Arabidopsis* and Yeast.

Further, these lncRNAs were also scanned against the genomes of five birds including Barn Owl, Mallard Duck, Rifleman, Rock Pigeon and Turkey selected randomly from the Avian Phylogenetic Database^37^. Most of the 100% similarity matches were obtained against Turkey (14 matches), followed by Mallard Duck (6), Barn owl (3) and Rock Pigeon (2). While there were no 100% similarity matches against Rifleman. All the sequences with 100% similarity were located in non-coding regions of the respective bird.

### Validation by RT-PCR

**Figure 5:**
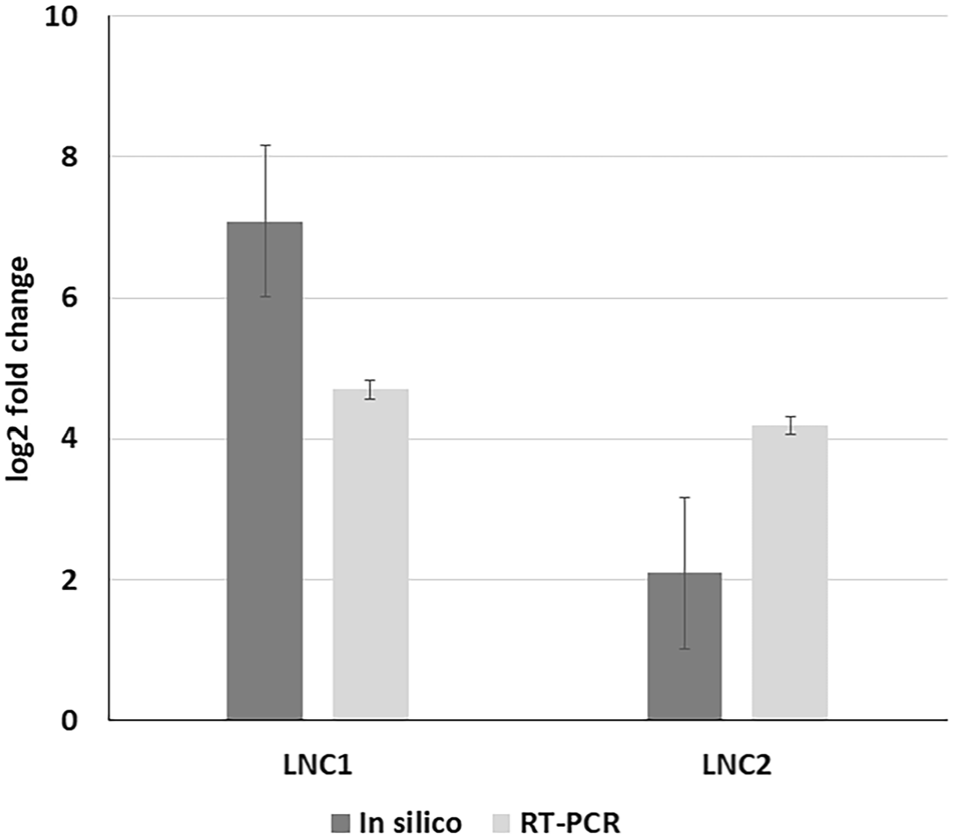
Figure showing the bar graphs representing the expression values of the two lncRNAs in the in-silico analysis and validation study (RT-PCR).

An experiment was conducted (mentioned in “Methods”) and RNA was extracted from the Harderian gland of White Leghorn and two lncRNAs were validated further for their expression. The expression was found to be in concordance with the expression observed in the in-silico analysis (Fig. 5).

## Discussion

Newcastle Disease is a serious threat to the global poultry industry. Previous studies show that Leghorn chicken breed has poor disease resistance and susceptible to NDV, while the Fayoumi breed is resistant and has comparatively better disease resistance. Long non coding RNAs (lncRNAs) contribute in various biological functions. In this study we have identified lncRNAs which were differentially expressing during NDV infection in Leghorn and Fayoumi breeds of *Gallus gallus*. Identification of lncRNAs in NDV resistance and susceptibility will have a big impact on poultry industry. These lncRNAs could also have clinical significance. However, the traditional methods to treat NDV were limited.

There were more differentially expressed genes and lncRNAs in Leghorn in initial stages and Fayoumi in latter stages. This shows that although there were more genes expressed initially in Leghorn, it lacked genes at latter stages of infection, while Fayoumi initially had lesser genes but eventually expressed more genes at latter stages, which could be the reason for clearance of viral load in Fayoumi.

From the Gene ontology analysis of DEGs, we can observe that there were more number of GO terms in Fayoumi than in Leghorn, across all the time points. Although, Leghorn showed a greater number of pathways initially, there were not many GO terms and no proper immune related pathways at latter stages of infection (10 DPC). On the other hand, Fayoumi showed immune pathway related GO terms across all time points (2, 6 and 10). In case of DElncRNAs, there was a same trend as DEGs with Fayoumi showing more number of GO terms than Leghorn. In Leghorn, at 10 DPC, there were no GO terms identified. Thus, we assume that the presence of immune related pathways across could be the reason for the Fayoumi to be comparatively resistant than Leghorn.

The gene co-expression network classifies the sets of co-ordinately expressed genes and lncRNAs into modules. The modules state that strongly co-related group of genes and lncRNAs are likely to be functionally associated. The co-expression analysis had revealed more number of interaction modules at Leghorn 6 DPC and Fayoumi 10 DPC. Although there were less number of genes and modules in Fayoumi at initial point of infection, it effectively clears the viral load from the blood by 6 DPC and gets rid of the infection by 10 DPC. While in Leghorn, there were more number of genes and pathways at initial point of infection, but the absence of proper pathways at latter stage of infection might be reason for lack of clearance of the viral load, making the bird get severe infection by 10 DPC and resulting in the death.

From the functional analysis of lncRNAs through co-expression, we observed that, in Leghorn, initially at 2 DPC, lncRNAs were co-expressing with the genes that were involved in immune related pathways, while at 6 DPC, most of the lncRNAs were found to be co-expressing with genes involved in metabolic pathways and a very few in immune related signalling pathways and at 10 DPC, there were only few co-expressing genes which were of unknown pathways. On the other hand, in Fayoumi, initially at 2 DPC, lncRNAs were co-expressing with comparatively less immune related genes than Leghorn, while at 6 DPC, there were more number of immunity and signalling pathway related co-expressing genes and at 10 DPC, unlike Leghorn, there were more number of immune related genes co-expressing with the lncRNAs. Apart from this, in Leghorn 2 DPC, Fayoumi 2 DPC and Fayoumi 6 DPC, all of the co-expression interactions included both lncRNAs and co-expressing genes getting up regulated. While in Leghorn 6 DPC, Leghorn 10 DPC and Fayoumi 10 DPC about 42%, 40% and 33% of interactions respectively included down regulated lncRNAs up regulating the co-expressing genes. From this information, we assume that in Leghorn, there were very few lncRNAs which were co-expressing with the genes that were involved in immune related pathways than Fayoumi, which could be the reason behind Leghorn being susceptible to NDV than Fayoumi. In Fayoumi, there were more number of lncRNAs co-expressing with genes involved in immune-related pathways, enabling it to express required genes and clear the viral load efficiently. Thus, we assume that the presence of the lncRNAs up regulating the genes involved in immune related pathways could help Leghorn becoming resistant to NDV.

We also predicted the interactions of the DEGs with transcription factors and microRNAs. Here, we observed a similar trend of interactions, with higher number of interactions at initial stages of infection and lesser number at latter stages in Leghorn and vice versa in Fayoumi. A further insight is needed in this regard to determine the exact interactions of the DEGs with the transcription factors and microRNAs.

From QTL analysis, we observed that genes were related to health QTLs only in Leghorn 6 DPC. We also observed that, at all the conditions, most of the DEGs i.e. 78% were associated with Production QTLs, 15% with Exterior QTL, 3% with Reproduction QTL and 2% each with Health QTL and Physiology QTL. Production QTLs were found in all the six conditions, Exterior QTLs and Reproduction QTLs were found in only Leghorn 6 DPC and Fayoumi 10 DPC, while Health QTLs and Physiology QTLs were found only in Leghorn 6 DPC.

From this, we observed that although there were genes and pathways expressed in Leghorn at the initial stage of infection, there were no immune related genes and pathways expressed at the latter stage, which makes it comparatively susceptible than Fayoumi. While the Fayoumi had fewer genes and pathways at the initial stages and more at the latter stage. Immune related pathways were observed across all the time points in Fayoumi, while there were no such pathways in Leghorn. The lncRNAs were also observed to be following the same trend as genes. Thus, we assume that the lncRNAs in Fayoumi that were co-expressing with the immune related genes might be the reason for the expression of genes and pathways. The molecular pathways, differentially expressed genes and lncRNAs identified will also be helpful in identifying the mechanism involved in NDV resistance/susceptibility and developing the strategies for augmenting resistance against NDV in Leghorn and Fayoumi breeds of *Gallus gallus*.

## Conclusion

Long non-coding RNAs (lncRNAs) are a family of non-coding regulatory RNAs which play a vital role in various biological processes, including host responses against different pathogens. Recent advancements in the sequencing technology and computational algorithms enable the identification and characterization of lncRNAs. The lncRNAs were less known in host responses against viruses especially in Leghorn and Fayoumi breeds of *Gallus gallus domesticus*. From the previous studies, we learnt that Fayoumi is more resistant than Leghorn, because of several immune related genes and pathways that were specifically expressed in Fayoumi. In this study we identified lncRNAs that were differentially expressing and regulating various genes in Harderian gland during host response against Newcastle disease virus challenge. We observed that there were more number of long non-coding RNAs expressed in Fayoumi that were co-expressing with the immune pathway related genes, while there were very few such lncRNAs in Leghorn. From this we assume that, these lncRNAs might be enhancing the expression of those genes which in turn making the Fayoumi breed more resistant than Leghorn. Future studies need to be done to unravel the mechanism through which the long non-coding RNAs regulate the genes.

## Methods

### Transcriptomic data collection

Owing to the recent widespread application of high-throughput RNA sequencing and computational pipelines, lncRNAs can now be predicted in diverse animal species to gain novel insights into their role in regulation of cellular processes. Harderian gland transcriptome data (94 datasets of approx. size 422 GB) of Leghorn and Fayoumi breeds of *Gallus gallus domesticus* was obtained from EBI-ENA database (Accession number—PRJEB22672). This data was submitted by Iowa State University. First, they collected the Harderian gland from challenged and non-challenged birds from each line at 2, 6, and 10 days-post-challenge (DPC). Then they isolated high quality RNA from the tissue and constructed two cDNA libraries from each RNA isolate (technical replicates) and sequenced using Illumina HiSeq 2500^10^. These 94 RNA-Seq data samples were of Harderian gland tissue from challenged and non-challenged birds at 2, 6, and 10 days-post-challenge (DPC) of Leghorn and Fayoumi breeds of *Gallus gallus*. The detailed information of the samples was included in Supplementary Table S11.

### Computational pipeline for the identification of lncRNAs

**Figure 6:**
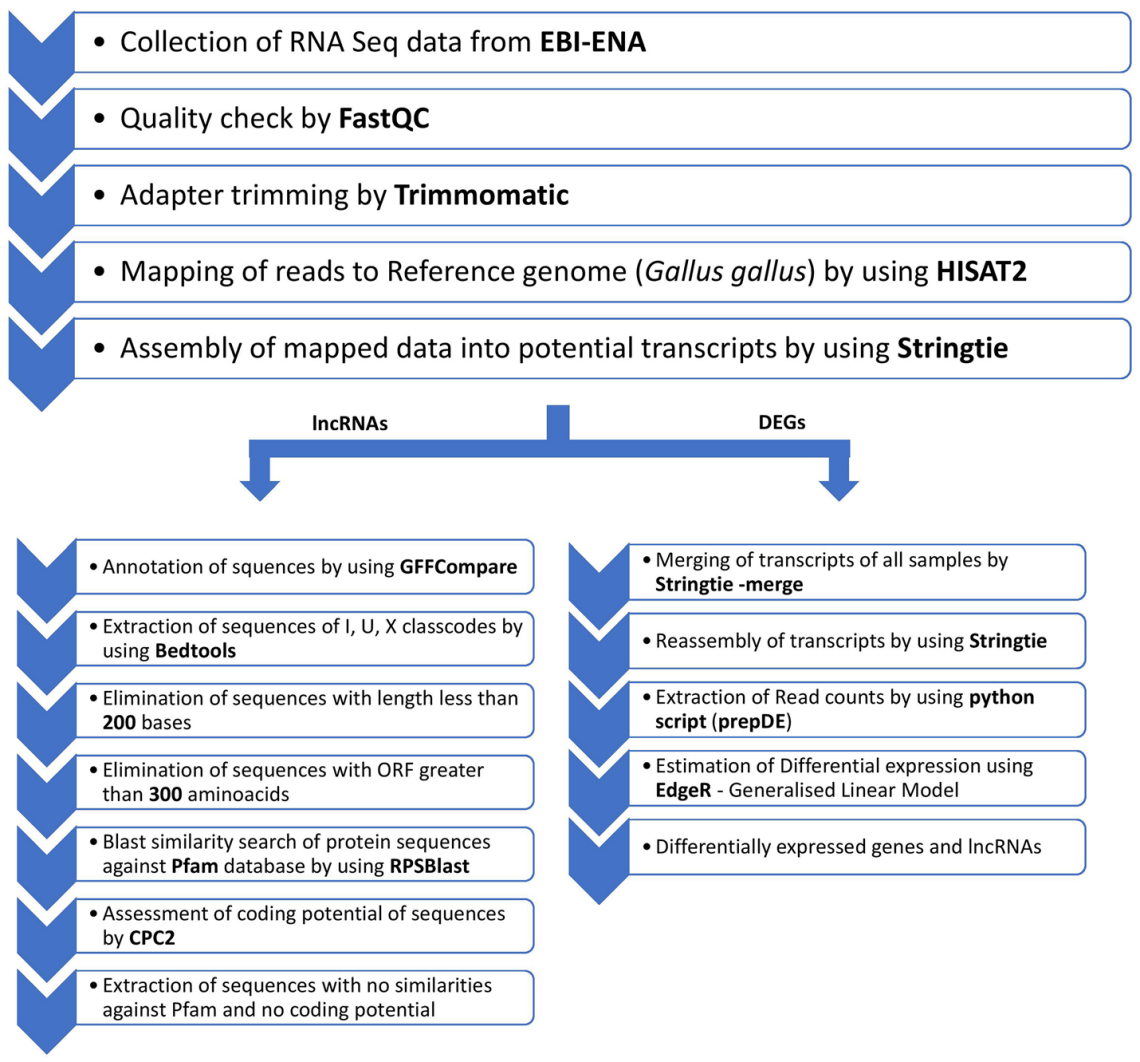
Overview of pipeline for the identification of long non-coding RNAs and differentially expressed genes.

A computational pipeline was developed as shown in Fig. 6, for identification of the lncRNAs. The differential expression analysis was performed by using latest version tools than those used in the original work^10^. The quality of the downloaded samples was analysed by using FastQC tool v0.11.8^11^. The results were merged using MultiQC tool v1.7^13^. After quality check adapter trimming was performed by using Trimmomatic tool v0.38^12^. After adapter trimming, quality was reanalysed by using FastQC tool v0.11.8^11^.

### Differential expression analysis

Trimmed sequences were mapped against the genome of *Gallus gallus* (GRCg6a) as reference by using Hisat2 tool v2.1.0^14^. Mapping was performed separately for each sample. The mapped sequences were then assembled into transcripts by using Stringtie tool v2.0.2^16^ and reference annotation file (GRCg6a). The output of Stringtie is written in Gene Transfer Format (.gtf) format. The transcripts of all the samples were merged in a single file in same format by using Stringtie merge function. Stringtie was again executed with initially mapped BAM files as input and the merged file as reference. This step was required to produce more accurate abundance estimations as per new Tuxedo pipeline^15^. Then the read counts were extracted by using python script provided with Stringtie. The gene count matrix was used to estimate the differential expression of genes by using edgeR tool^17^ by using Generalised linear model (GLM) as used previously^10^. The genes with adjusted P Value (FDR) less than 0.05 were selected as significantly differentiating genes. The differentially expressing lncRNAs were identified by using the same protocol by using annotation file (gff) containing sequences related to lncRNAs.

Using InteractiVenn^18^, we plotted the venn diagrams showing the unique and common DEGs of Leghorn and Fayoumi separately at each time point and between different time points of Leghorn and Fayoumi separately. The localization of these DEGs and DElncRNAs was plotted by using Circos^19^. Simple sequence repeats (SSRs) in DEGs and DElncRNAs were identified by using MISA^20^ standalone tool v2.1 separately for each DPC. The chromosomal localization of these SSRs was plotted by using Circos^19^.

### Genome-wide identification of lncRNAs in *Gallus gallus*

GFFCompare v0.11.2 tool^21^ was used to assign a class code for each transcript^21^ basing on the type of transcript of all the six conditions separately. The coordinates of transcripts of class codes I (intronic lncRNA), U (intergenic lncRNA) and X (antisense lncRNA) were extracted in bed format and sequences were extracted from the reference genome (GRCg6a) using Bedtools v2.28^22^. The extracted sequences were subjected to length filter and sequences with length less than 200 were discarded. Then OrfPredictor standalone tool^38^ was used for predicting the protein sequences. DNA sequences with ORFs of length greater than 300 bases in any of the six frames of translation were discarded. The remaining sequences were scanned against Pfam database^39^ by using RPSBlast^24^ with e-value 1e-3 and coding potential was calculated of corresponding nucleotide sequences by using Coding Potential Calculator v2^40^. The sequences with noORF, no hits against Pfam database and having noncoding tag in CPC2 were extracted and considered as potential lncRNAs. The localization of extracted lncRNAs was done by using Phenogram tool^23^. Apart from this, the extracted lncRNAs were searched for similarities against several databases like noncode database^25^ of *Gallus gallus*, transfer RNA database^26^, Silva rRNA database^27^, 5 s rRNA database^28^, mirBase^29^ by using standalone BlastN^24^.

### GO enrichment analysis

Gene ontology analysis of the DEGs and DElncRNAs was performed by using Blast2GO Basic standalone version 5^35^. The sequences were extracted from reference genome (GRCg6a) in fasta format using Bedtools^22^. The extracted sequences were given as input to Blast2GO. Several steps including Blast against non-redundant (nr) protein database with taxonomy filter of Birds (Aves, ID: 8782), EMBL-EBI Interpro, GO mapping (version 2020_06) and annotation were performed. The charts and graphs were plotted for Biological pathways, Molecular function and Cellular component. Pathway maps were loaded from online KEGG database^42^ through the integrated KEGG pathway tool.

### Co-expression analysis of lncRNAs

The functional annotation of the lncRNAs was performed by the co-expression analysis of these transcripts with the differentially expressed genes using WGCNA (Weighted Gene Correlation Network Analysis)^30^. It is an R-based standalone tool. Co-expression analysis was performed separately for each time point. The interactions between lncRNAs and genes with weight greater than 0.01 were further processed and visualized by using Cytoscape^31^ in form of networks. For better visualization, pathways of genes obtained through GO enrichment analysis were taken and the genes which were involved in Biological processes—Cellular Process (GO:0009987), response to stress (GO:0006950) and Immune system development (GO:0002520) were considered initially. Although the network containing all the genes and lncRNAs was visualized later.

### Cis and Trans regulation

The regulation of the genes by lncRNAs can be both Cis and Trans-regulation. Cis regulation is where the gene and lncRNA regulating it were on same chromosome and while in Trans-regulation, the gene and lncRNA were on different chromosome^41^. The chromosomal localization of the DEGs and the lncRNAs interacting with them was used to find the type of regulation between them.

### Functional analysis of lncRNAs

We used co-expression analysis for predicting the functions of lncRNAs. For this, we predicted the functions of the genes that were co-expressing with these lncRNA. The list of genes co-expressing with each of the lncRNAs obtained from the WGCNA analysis was taken and the KEGG pathways for each of the gene were obtained. These pathways were then assigned to the respective lncRNA.

### QTL (quantitative trait locus) analysis

The database of QTLs (Quantitative Trait Locus) of *Gallus gallus* was downloaded from the Animal QTL Database^36^ in gff file format, which contains the details of all the annotated QTLs of *Gallus gallus*. The coordinates of each QTL, differentially expressed genes and long non-coding RNAs were taken and QTLs that contain these differentially expressed genes were determined by using a python script.

### Interactions between DEGs and transcription factors

The 5′ upstream untranslated regions of 5 KB size of all the differentially expressed genes were extracted. These sequences were subjected for motif discovery by using MEME standalone tool^32^ with a limit of 10 motifs. The obtained motifs were then subjected to comparison against JASPER 2018 Vertebrates database using Tomtom standalone tool^32^ and the potential transcription factors binding to these motifs (genes) were identified. As the obtained transcription factors were of different species, these were searched against transcription factors of *Gallus gallus* downloaded from Animal Transcription factor database^18^ to obtain transcription factors of *Gallus gallus*.

### Interactions between DEGs and microRNAs

The microRNAs interacting with DEGs were obtained by using miRDB^34^, an online database for miRNA target prediction. This uses MirTarget tool, which was developed by analysing thousands of miRNA-target interactions from high-throughput sequencing experiments. This takes the gene name as input and gives a list of the potential miRNAs interacting with the gene. The top most miRNA, basing on Target score is selected as the potential miRNA interacting with the gene.

### Sequence similarity analysis

The extracted lncRNAs were analysed for sequence similarity by using Blast^24^ against the lncRNA data of all the available species on Noncode database^25^, including Human, Mouse, Cow, Rat, *C. elegans*, *Drosophila*, Zebrafish, *Arabidopsis*, Yeast, Chimp, Gorilla, Orangutan, Rhesus monkey, Opossum, Platypus and Pig. Further, these lncRNAs were also scanned against the genomes of five birds including Barn Owl, Peking duck, Rifleman, Rock Pigeon and Turkey selected randomly from the avian phylogenetic database^37^. All the extracted lncRNA of all the conditions were merged and searched using Blast^24^. The output was analysed and the hits with 100% similarity were considered to be similar.

### Validation studies

Animal experimentation was conducted with white leghorn chicken breed as a research material and was in accordance with Institute animal ethics committee guidelines (452/01/ab/CPCSEA). At 3 weeks of age, birds were challenged with lentogenic D58 strain of NDV (106 EID50) via intraocular route while the control group received PBS simultaneously. Harderian gland samples were collected aseptically at 2, 6 and 10 DPC. Total RNA was isolated and cDNA was synthesized using quantitect reverse transcriptase (Qiagen, Germany). Gene specific primers were designed targeting the two lncRNAs selected randomly and GAPDH (as housekeeping gene) (Table 8). SYBR green based qRT-PCR was performed to analyse the relative expression levels using 2^−∆∆CT^^43^.

**Table 8:** Details of the Oligonucleotides used in the validation study.

Gene	Forward (5′–3′)	Reverse (5′–3′)	Amplicon size (bp)	Annealing temperature (°C)
LNC 1	5′ ttggacacaggagaacagcttgag3'	5′ gggttgaagaggattgcgtttgg3'	247	60
LNC 2	5′ ttccgtcacgtatcttccttctcca 3'	5′ cgggatgatctgttgtgtgtggtagg 3'	217	58.3
GAPDH (NM_204305)	5′ agcacccgcatcaaagg 3'	5′ catcatcccagcgtcca 3'	263	60

### Ethics statement

Protocols concerning handling and care of birds during management and sample collection during the experiment were approved by Institute’s Animal Ethics Committee of ICAR-Central Avian Research Institute, Izatnagar, India (452/01/ab/CPCSEA) and were in accordance with guidelines Committee for the Purpose of Control and Supervision of Experiments on Animals, a statutory Committee, which is established under Chapter 4, Section 15(1) of the Prevention of Cruelty to Animals Act 1960, India. The research involved no human participants and also complies with Arrive3 guidelines.

## Supplementary Information

Supplementary Information 1.

Supplementary Information 2.

Supplementary Information 3.

Supplementary Information 4.

Supplementary Information 5.

Supplementary Information 6.

Supplementary Information 7.

Supplementary Information 8.

Supplementary Information 9.

Supplementary Information 10.

Supplementary Information 11.

Supplementary Information 12.

Supplementary Information 13.

Supplementary Information 14.

Supplementary Information 15.

Supplementary Information 16.

Supplementary Information 17.

Supplementary Information 18.

Supplementary Information 19.

## Abbreviations

lncRNA: Long non-coding RNA
DPC: Days post challenge
DEGs: Differentially expressed genes
DElncRNAs: Differentially expressed lncRNAs
NDV: Newcastle disease virus
