# Supplementary material for: Transcriptomic analysis to infer key molecular players involved during host response to NDV challenge in Gallus gallus (Leghorn & Fayoumi)
Source: Sci Rep. 2021 Apr 19;11:8486. doi: 10.1038/s41598-021-88029-6 (PMC8055681; doi:10.1038/s41598-021-88029-6)
Supplement: Supplementary file 3 — Supplementary Information 3. [file 41598_2021_88029_MOESM3_ESM.pdf]

Manuscript Title: Transcriptomic analysis to infer key molecular players involved during host response to NDV challenge in Gallus gallus (Leghorn & Fayoumi)

Authors: Vanamamalai Venkata Krishna[1], Priyanka Garg[1], Gautham Kolluri[2], Ravi Kumar Gandham[1], Itishree Jali[1], Shailesh Sharma[1*]

Affilation:

1. National Institute of Animal Biotechnology (NIAB), Opp. Journalist Colony, Near Gowlidoddi Extended Q City Road, Gachibowli Hyderabad, Telangana, India – 500032.
2. ICAR – Central Avian Research Institute, Izatnagar, Bareilly, Uttar Pradesh, India – 243122.

***Corresponding Author:** Dr. Shailesh Sharma, Scientist D, National Institute of Animal Biotechnology (NIAB), Opp. Journalist Colony, Near Gowlidoddi Extended Q City Road, Gachibowli, Hyderabad, Telangana, India – 500032

Email: shailesh.sharma@niab.org.in, haitoshailesh@gmail.com

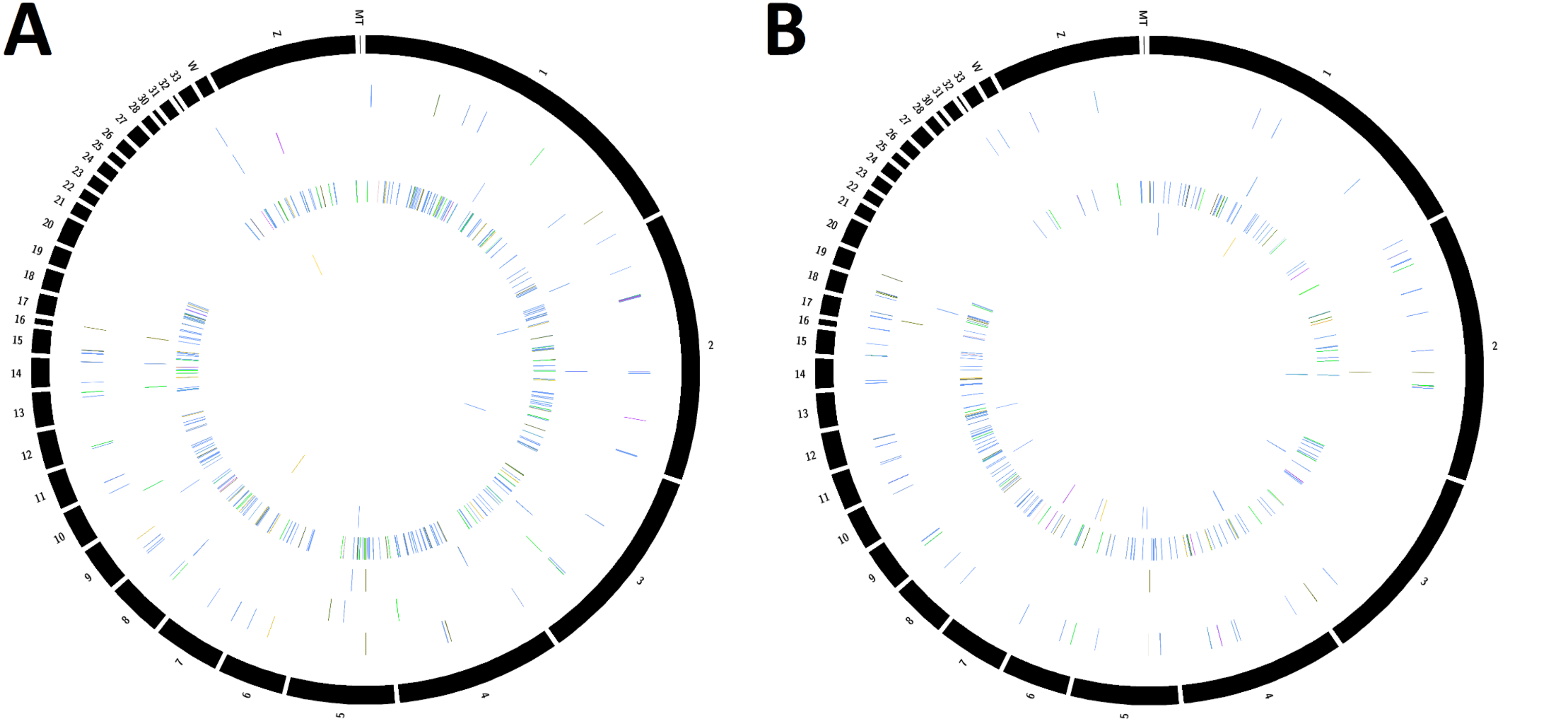

Supplementary Figure S3: Figure showing the circos plot of Simple sequence repeats obtained in differentially expressed genes (A) and differentially expressed lncRNAs (B) of Leghorn and Fayoumi. The main ideogram circle represents the chromosomes, each concentric circle represents the time point from outer to inner representing Fayoumi 10 DPC, Leghorn 10 DPC, Fayoumi 6 DPC, Leghorn 6 DPC, Fayoumi 2 DPC and Leghorn 2 DPC. While the different colours represent different SSR type (mono, di, tri, tetra, penta, hexanucleotide, compound microsatellites).
