# Supplementary material for: Transcriptomic analysis to infer key molecular players involved during host response to NDV challenge in Gallus gallus (Leghorn & Fayoumi)
Source: Sci Rep. 2021 Apr 19;11:8486. doi: 10.1038/s41598-021-88029-6 (PMC8055681; doi:10.1038/s41598-021-88029-6)
Supplement: Supplementary file 7 — Supplementary Information 7. [file 41598_2021_88029_MOESM7_ESM.pdf]

Manuscript Title: Transcriptomic analysis to infer key molecular players involved during host response to NDV challenge in Gallus gallus (Leghorn & Fayoumi)

Authors: Vanamamalai Venkata Krishna[1], Priyanka Garg[1], Gautham Kolluri[2], Ravi Kumar Gandham[1], Itishree Jali[1], Shailesh Sharma[1*]

Affilation:

1. National Institute of Animal Biotechnology (NIAB), Opp. Journalist Colony, Near Gowlidoddi Extended Q City Road, Gachibowli Hyderabad, Telangana, India – 500032.
2. ICAR – Central Avian Research Institute, Izatnagar, Bareilly, Uttar Pradesh, India – 243122.

***Corresponding Author:** Dr. Shailesh Sharma, Scientist D, National Institute of Animal Biotechnology (NIAB), Opp. Journalist Colony, Near Gowlidoddi Extended Q City Road, Gachibowli, Hyderabad, Telangana, India – 500032

Email: shailesh.sharma@niab.org.in, haitoshailesh@gmail.com

A:

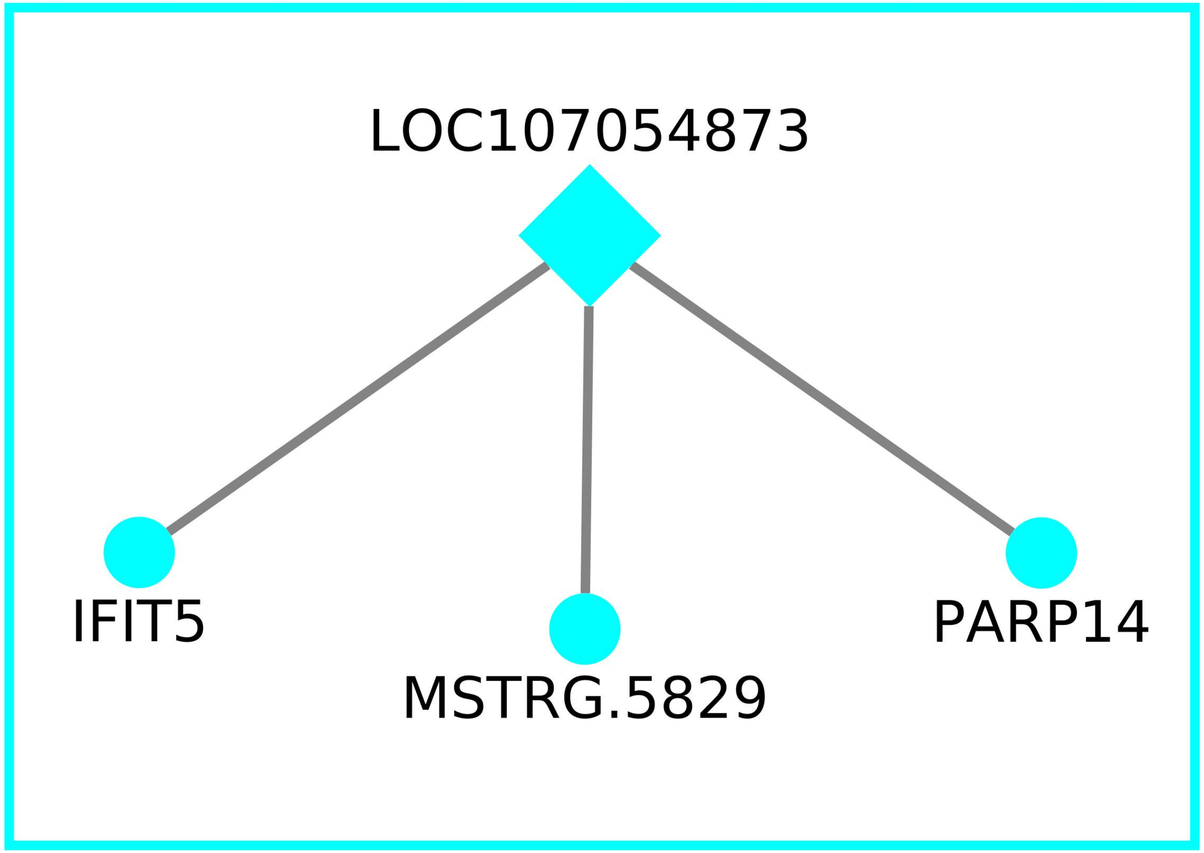

B:

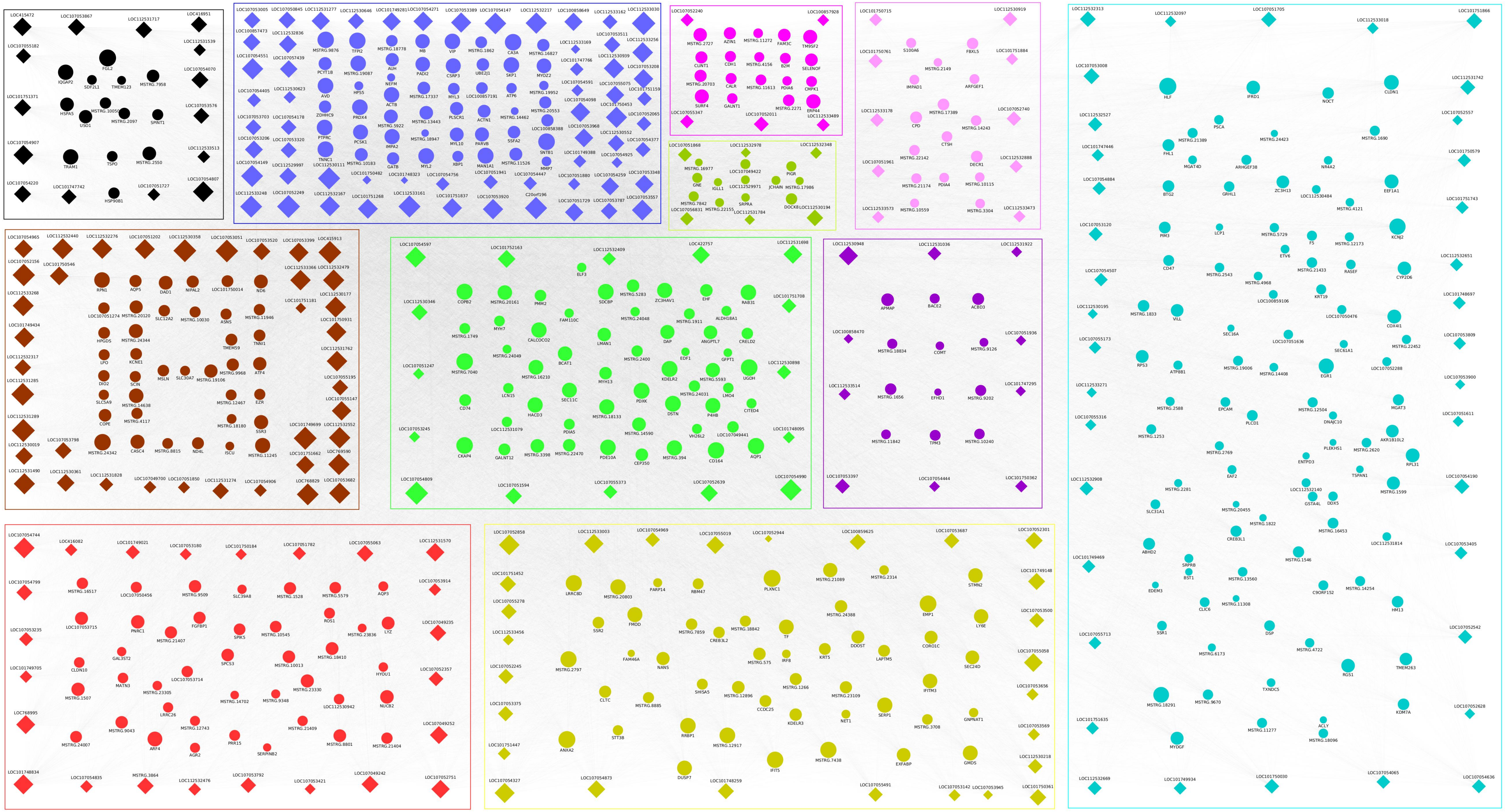

C:

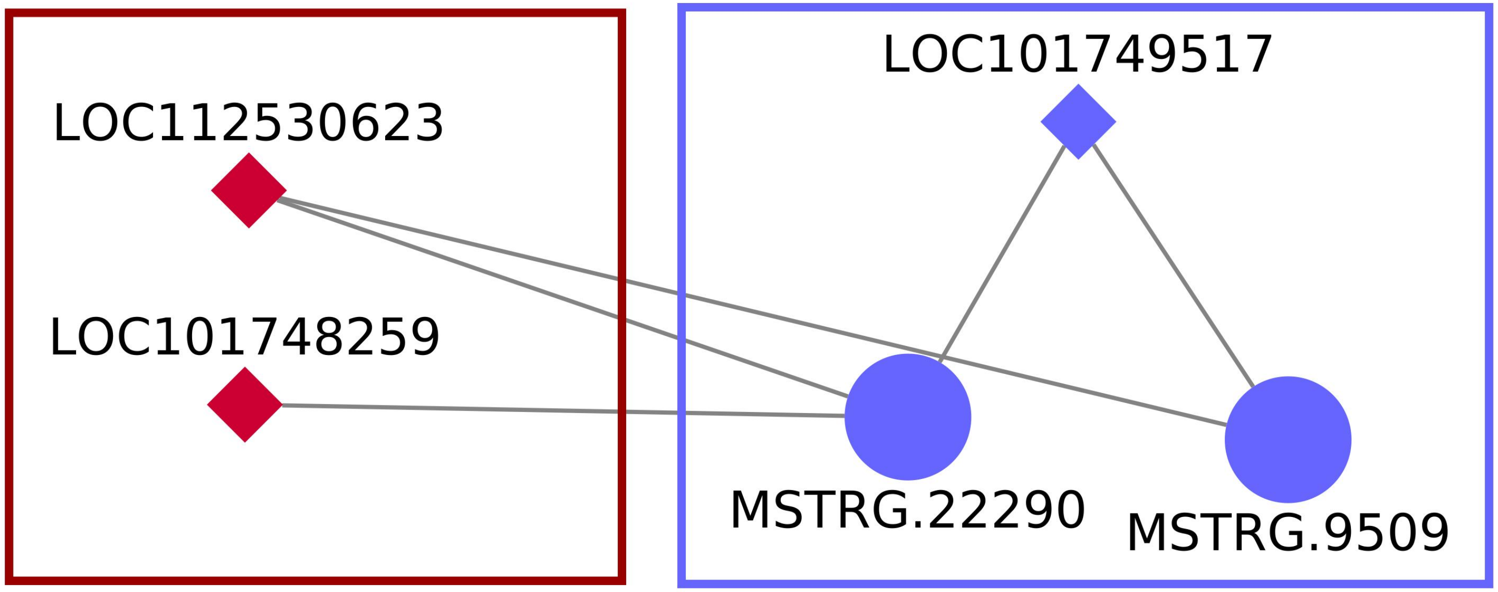
LOC112530623
LOC101748259
LOC101749517
MSTRG.22290
MSTRG.9509

D:

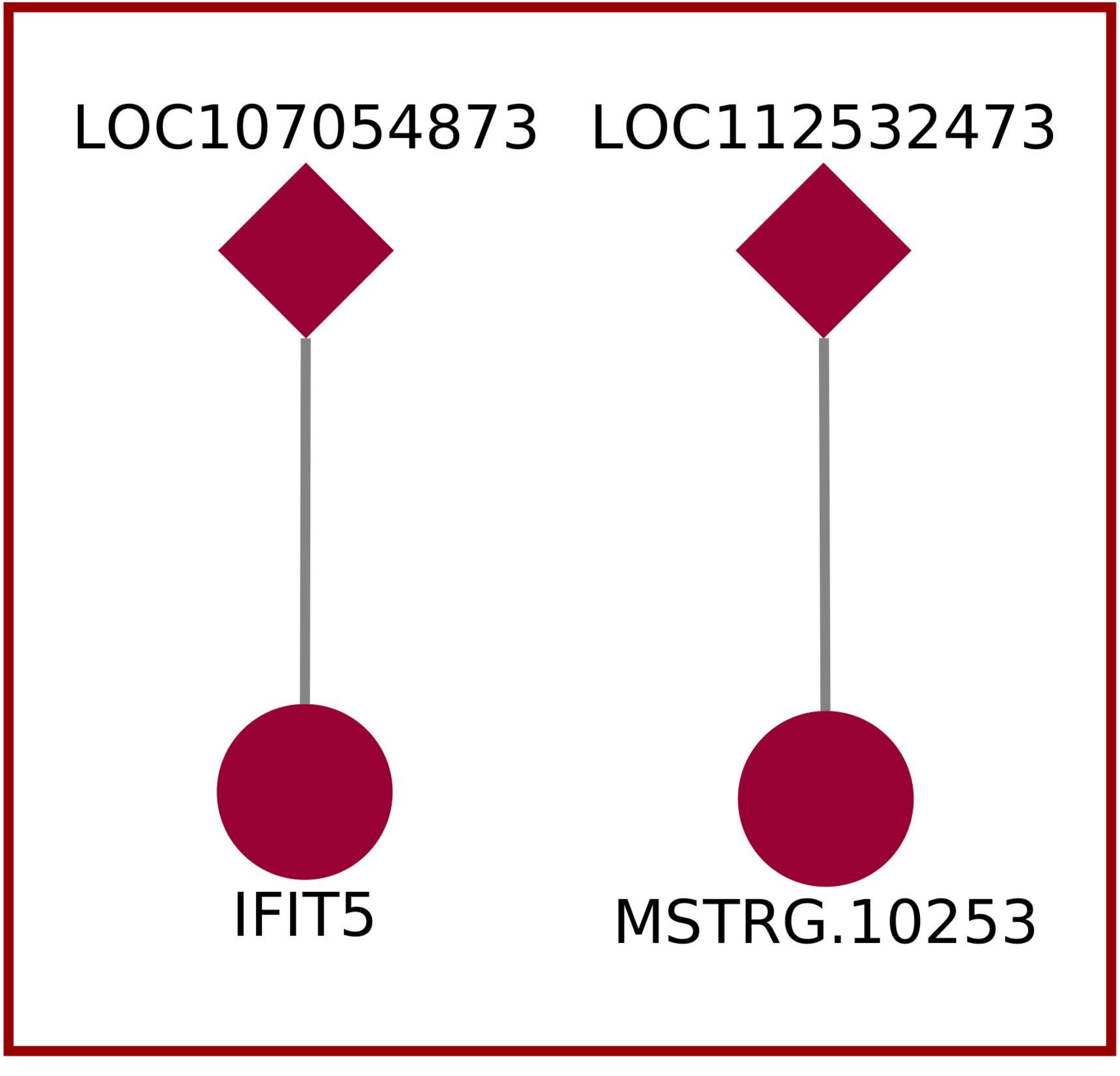

E:

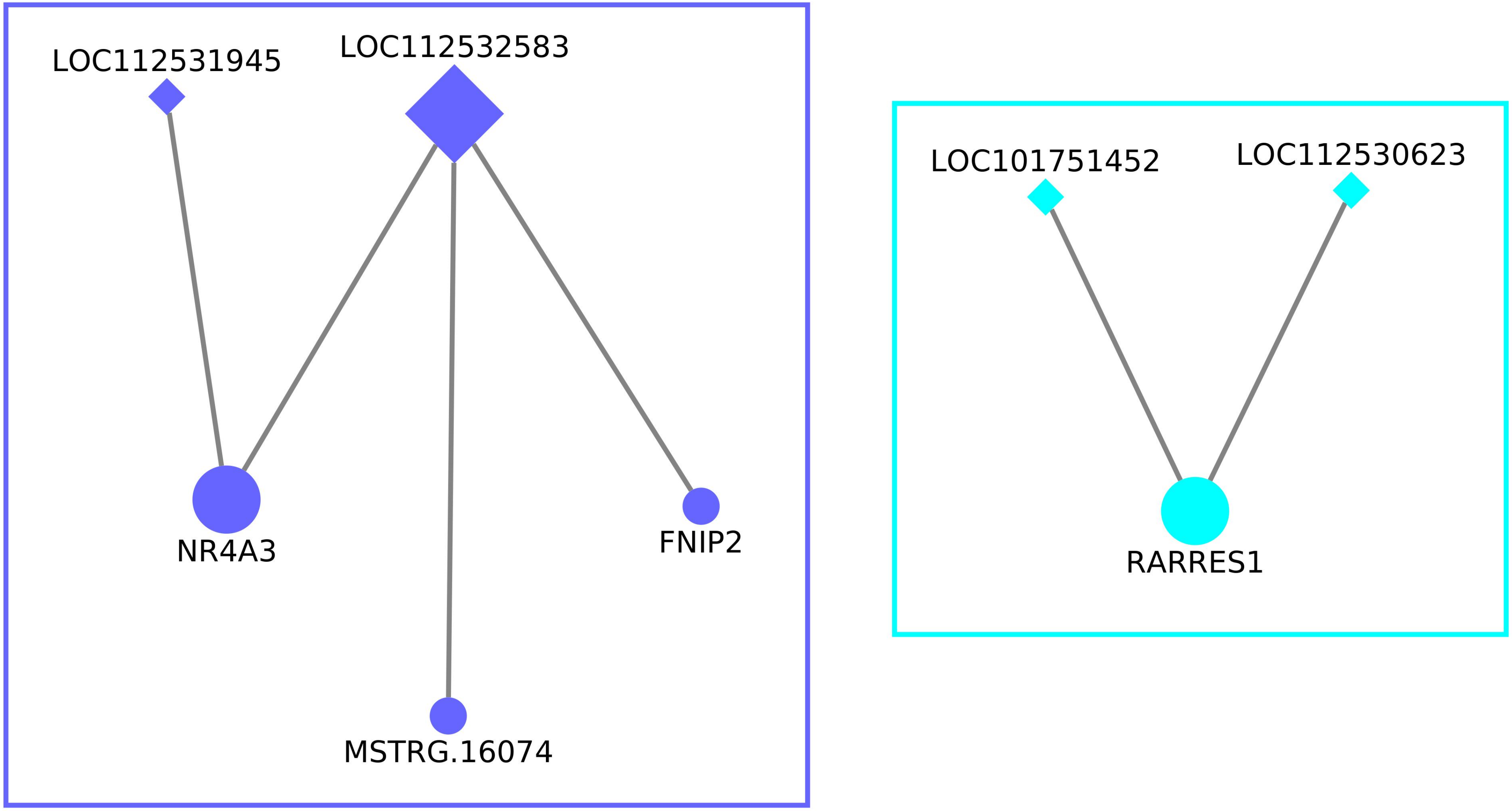
LOC112531945
LOC112532583
NR4A3
FNIP2
MSTRG.16074
LOC101751452
LOC112530623
RARRES1

F:

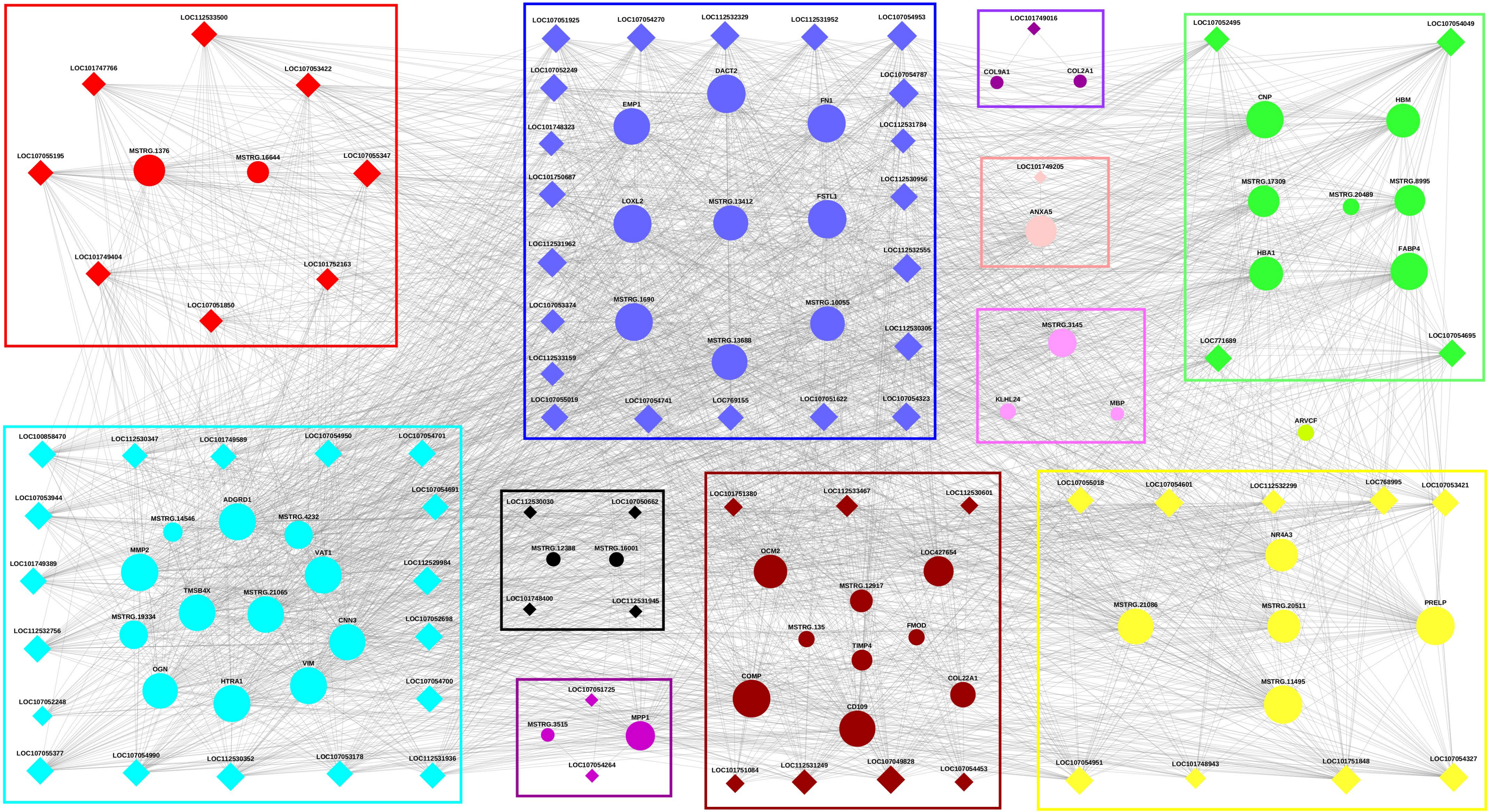

LOC112533500
LOC101747766
LOC107053422
LOC107055195
MSTRG.1376
MSTRG.16644
LOC107055347
LOC101749404
LOC101752163
LOC107051850
LOC107051925
LOC107054270
LOC112532329
LOC112531952
LOC107054953
LOC107052249
DACT2
LOC107054787
EMP1
FN1
LOC101748323
LOC112531784
LOC101750687
LOC112530956
LOXL2
MSTRG.13412
FSTL1
LOC112531962
LOC112532555
LOC107053374
MSTRG.1690
MSTRG.10055
LOC112530305
MSTRG.13688
LOC112533159
LOC107055019
LOC107054741
LOC769155
LOC107051622
LOC107054323
LOC101749016
COL9A1
COL2A1
LOC101749205
ANXA5
MSTRG.3145
KLHL24
MBP
LOC107052495
LOC107054049
CNP
HBM
MSTRG.17309
MSTRG.20489
MSTRG.8995
HBA1
FABP4
LOC771689
LOC107054695
ARVCF
LOC100858470
LOC112530347
LOC101749589
LOC107054950
LOC107054701
LOC107053944
LOC107054691
ADGRD1
MSTRG.14546
MSTRG.4232
MMP2
VAT1
LOC101749389
LOC112529984
TMSB4X
MSTRG.21065
MSTRG.19334
CNN3
LOC112532756
LOC107052698
OGN
VIM
HTRA1
LOC107054700
LOC107052248
LOC107055377
LOC107054990
LOC112530352
LOC107053178
LOC112531936
LOC112530030
LOC107050662
MSTRG.12388
MSTRG.16001
LOC101748400
LOC112531945
LOC107051725
MSTRG.3515
MPP1
LOC107054264
LOC101751380
LOC112533467
LOC112530601
OCM2
LOC427654
MSTRG.12917
MSTRG.135
FMOD
TIMP4
COMP
COL22A1
CD109
LOC101751084
LOC112531249
LOC107049828
LOC107054453
LOC107055018
LOC107054601
LOC112532299
LOC768995
LOC107053421
NR4A3
MSTRG.21086
MSTRG.20511
PRELP
MSTRG.11495
LOC107054951
LOC101748943
LOC101751848
LOC107054327

Supplementary Figure S7: Figure showing the Cytoscape figure showing networks of differentially expressed genes and long non-coding RNAs of all the Biological processes (GOs) of Leghorn 2 DPC (A), 6 DPC (B), 10 DPC (C) and Fayoumi 2 DPC (D), 6 DPC (E) and 10 DPC (F).
