# Supplementary material for: Transcriptomic analysis to infer key molecular players involved during host response to NDV challenge in Gallus gallus (Leghorn & Fayoumi)
Source: Sci Rep. 2021 Apr 19;11:8486. doi: 10.1038/s41598-021-88029-6 (PMC8055681; doi:10.1038/s41598-021-88029-6)
Supplement: Supplementary file 9 — Supplementary Information 9. [file 41598_2021_88029_MOESM9_ESM.pdf]

Manuscript Title: Transcriptomic analysis to infer key molecular players involved during host response to NDV challenge in Gallus gallus (Leghorn & Fayoumi)

Authors: Vanamamalai Venkata Krishna[1], Priyanka Garg[1], Gautham Kolluri[2], Ravi Kumar Gandham[1], Itishree Jali[1], Shailesh Sharma[1*]

Affilation:

1. National Institute of Animal Biotechnology (NIAB), Opp. Journalist Colony, Near Gowlidoddi Extended Q City Road, Gachibowli Hyderabad, Telangana, India – 500032.
2. ICAR – Central Avian Research Institute, Izatnagar, Bareilly, Uttar Pradesh, India – 243122.

***Corresponding Author:** Dr. Shailesh Sharma, Scientist D, National Institute of Animal Biotechnology (NIAB), Opp. Journalist Colony, Near Gowlidoddi Extended Q City Road, Gachibowli, Hyderabad, Telangana, India – 500032

Email: shailesh.sharma@niab.org.in, haitoshailesh@gmail.com

A:

SAMPLE	TRIMMOMATIC		
	Input	Survived	Survived%
ERX2181446	4261000	4213994	98.90
ERX2181447	12227000	12154243	99.40
ERX2181458	5546000	5414502	97.63
ERX2181459	5670000	5613752	99.01
ERX2181460	25062000	24604074	98.17
ERX2181461	21406000	21149047	98.80
ERX2181466	8357000	8270121	98.96
ERX2181467	3625000	3518019	97.05
ERX2181468	3671000	3627260	98.81
ERX2181469	18110000	17874750	98.70
ERX2181470	5181000	5136284	99.14
ERX2181471	3146000	3109494	98.84
ERX2181476	4155000	4101423	98.71
ERX2181477	20234000	19929974	98.50
ERX2181478	7510000	7416643	98.76
ERX2181479	9521000	9457380	99.33

B:

SAMPLE	TRIMMOMATIC		
	Input	Survived	Survived%
ERX2181436	27568000	27077574	98.22
ERX2181437	2258000	2205269	97.66
ERX2181438	4812000	4696639	97.60
ERX2181439	3818000	3784372	99.12
ERX2181440	7693000	7594934	98.73
ERX2181441	18898000	18698794	98.95
ERX2181448	15391000	15112236	98.19
ERX2181449	9831000	8888813	90.42
ERX2181450	2736000	2711312	99.10
ERX2181451	20882000	20602306	98.66
ERX2181456	9912000	9804798	98.92
ERX2181457	10696000	8452049	79.02
ERX2181472	7625000	7394965	96.98
ERX2181473	9363000	9239486	98.68
ERX2181474	11785000	8753162	74.27
ERX2181475	8406000	7937406	94.43

C:

SAMPLE	TRIMMOMATIC		
	Input	Survived	Survived%
ERX2181442	7066000	6985661	98.86
ERX2181443	3075000	3045736	99.05
ERX2181444	4281000	4005979	93.58
ERX2181445	5628000	5539281	98.42
ERX2181452	34332000	33825972	98.53
ERX2181453	7915000	7247030	91.56
ERX2181454	26583000	25806962	97.08
ERX2181455	15898000	15589896	98.06
ERX2181462	41859000	41379559	98.65
ERX2181463	5790000	5734624	99.04
ERX2181464	9138000	9054897	99.09
ERX2181465	2744000	2721449	99.18
ERX2181480	31660000	31269740	98.77
ERX2181481	10364000	8200777	79.13
ERX2181482	14496000	14058629	96.98
ERX2181483	10830000	10777957	99.52

D:

SAMPLE	TRIMMOMATIC		
	**Input**	**Survived**	**Survived%**
ERX2181484	33560000	33117038	98.68
ERX2181485	5372000	5328812	99.20
ERX2181490	11356000	11128980	98.00
ERX2181491	8119000	7914922	97.49
ERX2181492	25348000	25062166	98.87
ERX2181493	27957000	27258046	97.50
ERX2181500	37008000	36519048	98.68
ERX2181501	20292000	19957643	98.35
ERX2181506	7310000	7199451	98.49
ERX2181507	29522000	29204421	98.92
ERX2181510	28509000	28158041	98.77
ERX2181511	12325000	11265112	91.40
ERX2181524	25163000	23182380	92.13
ERX2181525	10330000	10244349	99.17
ERX2181528	21173000	20851466	98.48
ERX2181529	41638000	40782149	97.94

E:

SAMPLE	TRIMMOMATIC		
	Input	Survived	Survived%
ERX2181488	26864000	26536455	98.78
ERX2181489	22146000	21671518	97.86
ERX2181498	5935000	5873094	98.96
ERX2181499	10786000	10423669	96.64
ERX2181502	4133000	4084766	98.83
ERX2181503	9338000	9182643	98.34
ERX2181504	8066000	7092204	87.93
ERX2181505	10547000	10476101	99.33
ERX2181508	5462000	5399481	98.86
ERX2181509	17369000	17191249	98.98
ERX2181518	18049000	17887642	99.11
ERX2181519	6163000	6097518	98.94
ERX2181520	14902000	14743107	98.93
ERX2181521	13181000	13098236	99.37
ERX2181522	4712000	4672384	99.16
ERX2181523	8267000	8192583	99.10

F:

SAMPLE	TRIMMOMATIC		
	**Input**	**Survived**	**Survived%**
ERX2181486	3006000	2946946	98.04
ERX2181487	11344000	11218705	98.90
ERX2181494	3435000	3382899	98.48
ERX2181495	7611000	7555463	99.27
ERX2181496	16294000	15291735	93.85
ERX2181497	6499000	6394268	98.39
ERX2181512	7018000	6528208	93.02
ERX2181513	30144000	29830833	98.96
ERX2181514	3484000	3450456	99.04
ERX2181515	18125000	17888421	98.69
ERX2181516	3870000	3810404	98.46
ERX2181517	4569000	3471068	75.97
ERX2181526	33215000	32773506	98.67
ERX2181527	14593000	14351314	98.34

Supplementary Table S1: Trimmomatic analysis table of Leghorn 2 DPC (A), 6 DPC (B), 10 DPC (C) and Fayoumi 2 DPC (D), 6 DPC (E), 10 DPC (F) showing the total number of input reads, survived reads and percentage of survived reads.
