# Supplementary material for: Transcriptomic analysis to infer key molecular players involved during host response to NDV challenge in Gallus gallus (Leghorn & Fayoumi)
Source: Sci Rep. 2021 Apr 19;11:8486. doi: 10.1038/s41598-021-88029-6 (PMC8055681; doi:10.1038/s41598-021-88029-6)
Supplement: Supplementary file 10 — Supplementary Information 10. [file 41598_2021_88029_MOESM10_ESM.pdf]

**Manuscript Title:** Transcriptomic analysis to infer key molecular players involved during host response to NDV challenge in Gallus gallus (Leghorn & Fayoumi)

**Authors:** Vanamamalai Venkata Krishna[1], Priyanka Garg[1], Gautham Kolluri[2], Ravi Kumar Gandham[1], Itishree Jali[1], Shailesh Sharma[1*]

**Affilation:**

1. National Institute of Animal Biotechnology (NIAB), Opp. Journalist Colony, Near Gowlidoddi Extended Q City Road, Gachibowli Hyderabad, Telangana, India – 500032.
2. ICAR – Central Avian Research Institute, Izatnagar, Bareilly, Uttar Pradesh, India – 243122.

***Corresponding Author:** Dr. Shailesh Sharma, Scientist D, National Institute of Animal Biotechnology (NIAB), Opp. Journalist Colony, Near Gowlidoddi Extended Q City Road, Gachibowli, Hyderabad, Telangana, India – 500032

**Email:** shailesh.sharma@niab.org.in, haitoshailesh@gmail.com

**A:**

| Sample Name | Duplicates (%) | GC Content (%) | Total Sequences (Millions) |
|---|---|---|---|
| ERX2181446 | 47.0% | 46% | 4.2 |
| ERX2181447 | 57.1% | 47% | 12.2 |
| ERX2181458 | 48.2% | 46% | 5.4 |
| ERX2181459 | 48.2% | 47% | 5.6 |
| ERX2181460 | 63.8% | 48% | 24.6 |
| ERX2181461 | 58.8% | 47% | 21.1 |
| ERX2181466 | 52.9% | 46% | 8.3 |
| ERX2181467 | 46.4% | 47% | 3.5 |
| ERX2181468 | 46.4% | 47% | 3.6 |
| ERX2181469 | 74.2% | 49% | 17.9 |
| ERX2181470 | 42.8% | 47% | 5.1 |
| ERX2181471 | 36.7% | 48% | 3.1 |
| ERX2181476 | 46.2% | 47% | 4.1 |
| ERX2181477 | 60.9% | 44% | 19.9 |
| ERX2181478 | 49.6% | 46% | 7.4 |
| ERX2181479 | 57.3% | 47% | 9.5 |

**B:**

| Sample Name | Duplicates (%) | GC Content (%) | Total Sequences (Millions) |
|---|---|---|---|
| ERX2181436 | 75.9% | 45% | 27.1 |
| ERX2181437 | 39.6% | 45% | 2.2 |
| ERX2181438 | 49.3% | 46% | 4.7 |
| ERX2181439 | 43.3% | 47% | 3.8 |
| ERX2181440 | 59.1% | 50% | 7.6 |
| ERX2181441 | 64.5% | 50% | 18.7 |
| ERX2181448 | 69.9% | 49% | 15.1 |
| ERX2181449 | 77.5% | 47% | 8.9 |
| ERX2181450 | 38.6% | 47% | 2.7 |
| ERX2181451 | 75.3% | 45% | 20.6 |
| ERX2181456 | 53.3% | 47% | 9.8 |
| ERX2181457 | 68.1% | 44% | 8.5 |
| ERX2181472 | 51.2% | 45% | 7.4 |
| ERX2181473 | 57.7% | 47% | 9.2 |
| ERX2181474 | 73.6% | 44% | 8.8 |
| ERX2181475 | 69.5% | 47% | 7.9 |

**C:**

| Sample Name | Duplicates (%) | GC Content (%) | Total Sequences (Millions) |
|---|---|---|---|
| ERX2181442 | 52.5% | 48% | 7.0 |
| ERX2181443 | 38.1% | 46% | 3.0 |
| ERX2181444 | 53.5% | 49% | 4.0 |
| ERX2181445 | 59.9% | 48% | 5.5 |
| ERX2181452 | 74.4% | 47% | 33.8 |
| ERX2181453 | 62.9% | 45% | 7.2 |
| ERX2181454 | 77.6% | 48% | 25.8 |
| ERX2181455 | 65.1% | 48% | 15.6 |
| ERX2181462 | 71.2% | 48% | 41.4 |
| ERX2181463 | 51.3% | 47% | 5.7 |
| ERX2181464 | 62.0% | 51% | 9.1 |
| ERX2181465 | 52.1% | 48% | 2.7 |
| ERX2181480 | 58.8% | 47% | 31.3 |
| ERX2181481 | 64.7% | 48% | 8.2 |
| ERX2181482 | 80.5% | 48% | 14.1 |
| ERX2181483 | 58.6% | 50% | 10.8 |

**D:**

| Sample Name | Duplicates (%) | GC Content (%) | Total Sequences (Millions) |
|---|---|---|---|
| ERX2181484 | 67.9% | 50% | 33.1 |
| ERX2181485 | 59.2% | 50% | 5.3 |
| ERX2181490 | 77.5% | 48% | 11.1 |
| ERX2181491 | 56.8% | 49% | 7.9 |
| ERX2181492 | 70.8% | 49% | 25.1 |
| ERX2181500 | 76.5% | 47% | 36.5 |
| ERX2181501 | 74.9% | 46% | 20.0 |
| ERX2181506 | 50.1% | 48% | 7.2 |
| ERX2181507 | 54.3% | 47% | 29.2 |
| ERX2181510 | 66.7% | 50% | 28.2 |
| ERX2181511 | 76.7% | 51% | 11.3 |
| ERX2181524 | 84.6% | 48% | 23.2 |
| ERX2181525 | 62.4% | 47% | 10.2 |
| ERX2181528 | 66.4% | 48% | 20.9 |
| ERX2181529 | 84.9% | 46% | 40.8 |

**E:**

| Sample Name | Duplicates (%) | GC Content (%) | Total Sequences (Millions) |
|---|---|---|---|
| ERX2181488 | 62.4% | 48% | 26.5 |
| ERX2181489 | 72.6% | 45% | 21.7 |
| ERX2181498 | 38.2% | 44% | 5.9 |
| ERX2181499 | 53.9% | 46% | 10.4 |
| ERX2181502 | 40.3% | 49% | 4.1 |
| ERX2181503 | 71.2% | 44% | 9.2 |
| ERX2181504 | 59.2% | 47% | 7.1 |
| ERX2181505 | 61.0% | 50% | 10.5 |
| ERX2181508 | 40.9% | 48% | 5.4 |
| ERX2181509 | 59.2% | 44% | 17.2 |
| ERX2181518 | 74.0% | 46% | 17.9 |
| ERX2181519 | 43.1% | 46% | 6.1 |
| ERX2181520 | 62.8% | 46% | 14.7 |
| ERX2181521 | 61.1% | 44% | 13.1 |
| ERX2181522 | 37.3% | 47% | 4.7 |
| ERX2181523 | 48.1% | 46% | 8.2 |

**F:**

| Sample Name | Duplicates (%) | GC Content (%) | Total Sequences (Millions) |
|---|---|---|---|
| ERX2181486 | 36.5% | 48% | 2.9 |
| ERX2181487 | 49.7% | 50% | 11.2 |
| ERX2181494 | 32.2% | 48% | 3.4 |
| ERX2181495 | 41.6% | 48% | 7.6 |
| ERX2181496 | 65.6% | 46% | 15.3 |
| ERX2181497 | 40.8% | 45% | 6.4 |
| ERX2181512 | 48.7% | 47% | 6.5 |
| ERX2181513 | 54.8% | 47% | 29.8 |
| ERX2181514 | 35.3% | 47% | 3.5 |
| ERX2181515 | 56.7% | 46% | 17.9 |
| ERX2181516 | 39.4% | 48% | 3.8 |
| ERX2181517 | 45.4% | 44% | 3.5 |
| ERX2181526 | 53.4% | 47% | 32.8 |
| ERX2181527 | 50.2% | 44% | 14.4 |

**Supplementary Table S2:** MultiQC analysis table of Leghorn 2 DPC (A), 6 DPC (B), 10 DPC (C) and Fayoumi 2 DPC (D), 6 DPC (E), 10 DPC (F) showing the percentage of Duplicates, GC content and total sequences in millions.
