# Supplementary material for: Transcriptomic analysis to infer key molecular players involved during host response to NDV challenge in Gallus gallus (Leghorn & Fayoumi)
Source: Sci Rep. 2021 Apr 19;11:8486. doi: 10.1038/s41598-021-88029-6 (PMC8055681; doi:10.1038/s41598-021-88029-6)
Supplement: Supplementary file 11 — Supplementary Information 11. [file 41598_2021_88029_MOESM11_ESM.pdf]

Manuscript Title: Transcriptomic analysis to infer key molecular players involved during host response to NDV challenge in Gallus gallus (Leghorn & Fayoumi)

Authors: Vanamamalai Venkata Krishna[1], Priyanka Garg[1], Gautham Kolluri[2], Ravi Kumar Gandham[1], Itishree Jali[1], Shailesh Sharma[1*]

Affilation:

1. National Institute of Animal Biotechnology (NIAB), Opp. Journalist Colony, Near Gowlidoddi Extended Q City Road, Gachibowli Hyderabad, Telangana, India – 500032.
2. ICAR – Central Avian Research Institute, Izatnagar, Bareilly, Uttar Pradesh, India – 243122.

***Corresponding Author:** Dr. Shailesh Sharma, Scientist D, National Institute of Animal Biotechnology (NIAB), Opp. Journalist Colony, Near Gowlidoddi Extended Q City Road, Gachibowli, Hyderabad, Telangana, India – 500032

Email: shailesh.sharma@niab.org.in, haitoshailesh@gmail.com

A:

SAMPLE	HISAT2	STRINGTIE	
		Before merge	After merge
ERX2181446	94.08	135868	1153289
ERX2181447	94.55	219153	1153289
ERX2181458	92.80	154445	1153289
ERX2181459	94.38	164547	1153289
ERX2181460	92.68	289665	1153289
ERX2181461	93.03	264055	1153289
ERX2181466	95.03	182702	1153289
ERX2181467	92.43	127147	1153289
ERX2181468	93.17	128281	1153289
ERX2181469	90.91	204460	1153289
ERX2181470	94.63	161004	1153289
ERX2181471	93.47	134792	1153289
ERX2181476	92.82	136200	1153289
ERX2181477	94.62	254682	1153289
ERX2181478	93.94	176616	1153289
ERX2181479	94.07	195477	1153289

B:

**SAMPLE**	**HISAT2**	**STRINGTIE**	
		**Before merge**	**After merge**
ERX2181436	93.46	257358	1159889
ERX2181437	93.64	94480	1159889
ERX2181438	92.20	145075	1159889
ERX2181439	94.65	135166	1159889
ERX2181440	89.18	190508	1159889
ERX2181441	86.59	231786	1159889
ERX2181448	89.05	221073	1159889
ERX2181449	84.13	148122	1159889
ERX2181450	93.52	118561	1159889
ERX2181451	95.00	225530	1159889
ERX2181456	93.02	206508	1159889
ERX2181457	75.29	155553	1159889
ERX2181472	92.44	171845	1159889
ERX2181473	93.78	191637	1159889
ERX2181474	71.23	146867	1159889
ERX2181475	88.99	161862	1159889

C:

SAMPLE	HISAT2	STRINGTIE	
		Before merge	After merge
ERX2181442	92.59	173769	1176016
ERX2181443	95.36	122235	1176016
ERX2181444	84.81	132522	1176016
ERX2181445	91.46	141157	1176016
ERX2181452	91.38	289298	1176016
ERX2181453	87.08	149216	1176016
ERX2181454	90.04	259339	1176016
ERX2181455	89.99	237874	1176016
ERX2181462	93.49	334886	1176016
ERX2181463	94.13	155154	1176016
ERX2181464	87.95	169785	1176016
ERX2181465	90.47	87177	1176016
ERX2181480	92.86	317273	1176016
ERX2181481	73.54	172640	1176016
ERX2181482	87.71	171227	1176016
ERX2181483	88.70	210319	1176016

D:

SAMPLE	HISAT2	STRINGTIE	
		Before merge	After merge
ERX2181484	83.90	264468	1246313
ERX2181485	86.84	135623	1246313
ERX2181490	88.55	167861	1246313
ERX2181491	87.54	187023	1246313
ERX2181492	90.09	276310	1246313
ERX2181493	10.34	81918	1246313
ERX2181500	92.95	320491	1246313
ERX2181501	93.57	238326	1246313
ERX2181506	92.13	194345	1246313
ERX2181507	93.04	350964	1246313
ERX2181510	85.43	255338	1246313
ERX2181511	77.78	165546	1246313
ERX2181524	82.53	203249	1246313
ERX2181525	92.66	191375	1246313
ERX2181528	92.83	270705	1246313
ERX2181529	93.93	262808	1246313

E:

SAMPLE	HISAT2	STRINGTIE	
		Before merge	After merge
ERX2181488	91.63	321969	1217310
ERX2181489	92.47	259426	1217310
ERX2181498	94.68	172357	1217310
ERX2181499	91.57	222628	1217310
ERX2181502	91.58	155247	1217310
ERX2181503	94.20	159123	1217310
ERX2181504	77.70	146504	1217310
ERX2181505	87.62	185522	1217310
ERX2181508	91.46	175175	1217310
ERX2181509	95.03	250948	1217310
ERX2181518	94.69	224754	1217310
ERX2181519	95.03	174713	1217310
ERX2181520	94.31	226614	1217310
ERX2181521	96.28	195458	1217310
ERX2181522	94.44	167278	1217310
ERX2181523	94.85	200140	1217310

F:

SAMPLE	HISAT2	STRINGTIE	
		**Before merge**	**After merge**
ERX2181486	89.34	131704	1176712
ERX2181487	87.99	231185	1176712
ERX2181494	92.22	145303	1176712
ERX2181495	93.32	203499	1176712
ERX2181496	89.13	238136	1176712
ERX2181497	94.79	177638	1176712
ERX2181512	87.39	183214	1176712
ERX2181513	92.81	343038	1176712
ERX2181514	91.70	138737	1176712
ERX2181515	93.16	259441	1176712
ERX2181516	90.29	148450	1176712
ERX2181517	71.05	120636	1176712
ERX2181526	92.29	356358	1176712
ERX2181527	92.77	253433	1176712

Supplementary Table S3: HISAT2 and Stringtie analysis table of Leghorn 2 DPC (A), 6 DPC (B), 10 DPC (C) and Fayoumi 2 DPC (D), 6 DPC (E), 10 DPC (F) showing the percentage of mapping in HISAT2, number of transcripts obtained from stringtie before merging and after merging.
