# Supplementary material for: Transcriptomic analysis to infer key molecular players involved during host response to NDV challenge in Gallus gallus (Leghorn & Fayoumi)
Source: Sci Rep. 2021 Apr 19;11:8486. doi: 10.1038/s41598-021-88029-6 (PMC8055681; doi:10.1038/s41598-021-88029-6)
Supplement: Supplementary file 19 — Supplementary Information 19. [file 41598_2021_88029_MOESM19_ESM.pdf]

Manuscript Title: Transcriptomic analysis to infer key molecular players involved during host response to NDV challenge in Gallus gallus (Leghorn & Fayoumi)

Authors: Vanamamalai Venkata Krishna[1], Priyanka Garg[1], Gautham Kolluri[2], Ravi Kumar Gandham[1], Itishree Jali[1], Shailesh Sharma[1*]

Affilation:

1. National Institute of Animal Biotechnology (NIAB), Opp. Journalist Colony, Near Gowlidoddi Extended Q City Road, Gachibowli Hyderabad, Telangana, India – 500032.
2. ICAR – Central Avian Research Institute, Izatnagar, Bareilly, Uttar Pradesh, India – 243122.

***Corresponding Author:** Dr. Shailesh Sharma, Scientist D, National Institute of Animal Biotechnology (NIAB), Opp. Journalist Colony, Near Gowlidoddi Extended Q City Road, Gachibowli, Hyderabad, Telangana, India – 500032

Email: shailesh.sharma@niab.org.in, haitoshailesh@gmail.com

A:

Breed	Leghorn		Fayoumi	
**DPC**	**Control**	**Infected**	**Control**	**Infected**
**2 DPC**	6	10	8	8
**6 DPC**	8	8	8	8
**10 DPC**	8	8	8	6

B:

SAMPLE	EXP ID	AGE	GENDER	DISEASE	REPLICATE	DAY
SAMEA104305996	ERX2181436	27	Male	Normal	1	6
SAMEA104305997	ERX2181437	27	Male	Normal	2	6
SAMEA104305998	ERX2181438	27	Female	Normal	1	6
SAMEA104305999	ERX2181439	27	Female	Normal	2	6
SAMEA104306000	ERX2181440	27	Female	NCD	1	6
SAMEA104306001	ERX2181441	27	Female	NCD	2	6
SAMEA104306002	ERX2181442	31	Female	NCD	1	10
SAMEA104306003	ERX2181443	31	Female	NCD	2	10
SAMEA104306004	ERX2181444	31	Male	Normal	1	10
SAMEA104306005	ERX2181445	31	Male	Normal	2	10
SAMEA104306006	ERX2181446	23	Male	Normal	1	2
SAMEA104306007	ERX2181447	23	Male	Normal	2	2
SAMEA104306008	ERX2181448	27	Male	NCD	1	6
SAMEA104306009	ERX2181449	27	Male	NCD	2	6
SAMEA104306010	ERX2181450	27	Male	Normal	1	6
SAMEA104306011	ERX2181451	27	Male	Normal	2	6
SAMEA104306012	ERX2181452	31	Male	Normal	1	10
SAMEA104306013	ERX2181453	31	Male	Normal	2	10

SAMEA104306014	ERX2181454	31	Female	Normal	1	10
SAMEA104306015	ERX2181455	31	Female	Normal	2	10
SAMEA104306016	ERX2181456	27	Female	NCD	1	6
SAMEA104306017	ERX2181457	27	Female	NCD	2	6
SAMEA104306018	ERX2181458	23	Female	NCD	1	2
SAMEA104306019	ERX2181459	23	Female	NCD	2	2
SAMEA104306020	ERX2181460	23	Female	NCD	1	2
SAMEA104306021	ERX2181461	23	Female	NCD	2	2
SAMEA104306022	ERX2181462	31	Male	NCD	1	10
SAMEA104306023	ERX2181463	31	Male	NCD	2	10
SAMEA104306024	ERX2181464	31	Male	NCD	1	10
SAMEA104306025	ERX2181465	31	Male	NCD	2	10
SAMEA104306026	ERX2181466	23	Female	NCD	1	2
SAMEA104306027	ERX2181467	23	Female	NCD	2	2
SAMEA104306028	ERX2181468	23	Female	Normal	1	2
SAMEA104306029	ERX2181469	23	Female	Normal	2	2
SAMEA104306030	ERX2181470	23	Male	NCD	1	2
SAMEA104306031	ERX2181471	23	Male	NCD	2	2
SAMEA104306032	ERX2181472	27	Female	Normal	1	6
SAMEA104306033	ERX2181473	27	Female	Normal	2	6
SAMEA104306034	ERX2181474	27	Male	NCD	1	6
SAMEA104306035	ERX2181475	27	Male	NCD	2	6
SAMEA104306036	ERX2181476	23	Male	NCD	1	2
SAMEA104306037	ERX2181477	23	Male	NCD	2	2
SAMEA104306038	ERX2181478	23	Male	Normal	1	2
SAMEA104306039	ERX2181479	23	Male	Normal	2	2

SAMEA104306040	ERX2181480	31	Female	Normal	1	10
SAMEA104306041	ERX2181481	31	Female	Normal	2	10
SAMEA104306042	ERX2181482	31	Female	NCD	1	10
SAMEA104306043	ERX2181483	31	Female	NCD	2	10

C:

SAMPLE	EXP ID	AGE	GENDER	DISEASE	REPLICATE	DAY
SAMEA104306044	ERX2181484	23	Male	Normal	1	2
SAMEA104306045	ERX2181485	23	Male	Normal	2	2
SAMEA104306046	ERX2181486	31	Female	Normal	1	10
SAMEA104306047	ERX2181487	31	Female	Normal	2	10
SAMEA104306048	ERX2181488	27	Male	NCD	1	6
SAMEA104306049	ERX2181489	27	Male	NCD	2	6
SAMEA104306050	ERX2181490	23	Female	NCD	1	2
SAMEA104306051	ERX2181491	23	Female	NCD	2	2
SAMEA104306052	ERX2181492	23	Female	Normal	1	2
SAMEA104306053	ERX2181493	23	Female	Normal	2	2
SAMEA104306054	ERX2181494	31	Male	Normal	1	10
SAMEA104306055	ERX2181495	31	Male	Normal	2	10
SAMEA104306056	ERX2181496	31	Male	NCD	1	10
SAMEA104306057	ERX2181497	31	Male	NCD	2	10
SAMEA104306058	ERX2181498	27	Female	NCD	1	6
SAMEA104306059	ERX2181499	27	Female	NCD	2	6
SAMEA104306060	ERX2181500	23	Female	Normal	1	2
SAMEA104306061	ERX2181501	23	Female	Normal	2	2
SAMEA104306062	ERX2181502	27	Female	Normal	1	6
SAMEA104306063	ERX2181503	27	Female	Normal	2	6

SAMEA104306064	ERX2181504	27	Male	Normal	1	6
SAMEA104306065	ERX2181505	27	Male	Normal	2	6
SAMEA104306066	ERX2181506	23	Female	NCD	1	2
SAMEA104306067	ERX2181507	23	Female	NCD	2	2
SAMEA104306068	ERX2181508	27	Male	NCD	1	6
SAMEA104306069	ERX2181509	27	Male	NCD	2	6
SAMEA104306070	ERX2181510	23	Male	NCD	1	2
SAMEA104306071	ERX2181511	23	Male	NCD	2	2
SAMEA104306072	ERX2181512	31	Female	NCD	1	10
SAMEA104306073	ERX2181513	31	Female	NCD	2	10
SAMEA104306074	ERX2181514	31	Female	Normal	1	10
SAMEA104306075	ERX2181515	31	Female	Normal	2	10
SAMEA104306076	ERX2181516	31	Male	Normal	1	10
SAMEA104306077	ERX2181517	31	Male	Normal	2	10
SAMEA104306078	ERX2181518	27	Male	Normal	1	6
SAMEA104306079	ERX2181519	27	Male	Normal	2	6
SAMEA104306080	ERX2181520	27	Female	NCD	1	6
SAMEA104306081	ERX2181521	27	Female	NCD	2	6
SAMEA104306082	ERX2181522	27	Male	Normal	1	6
SAMEA104306083	ERX2181523	27	Male	Normal	2	6
SAMEA104306084	ERX2181524	23	Male	NCD	1	2
SAMEA104306085	ERX2181525	23	Male	NCD	2	2
SAMEA104306086	ERX2181526	31	Female	NCD	1	10
SAMEA104306087	ERX2181527	31	Female	NCD	2	10
SAMEA104306088	ERX2181528	23	Male	Normal	1	2
SAMEA104306089	ERX2181529	23	Male	Normal	2	2

Supplementary Table S11: Tables showing the number of samples (A), details of each sample of Leghorn (B) and Fayoumi (C) breeds.
